# AH-6809 mediated regulation of lung adenocarcinoma metastasis through NLRP7 and prognostic analysis of key metastasis-related genes

**DOI:** 10.3389/fphar.2024.1486265

**Published:** 2024-12-04

**Authors:** Xu Feng, Wei Wu, Feifei Liu

**Affiliations:** ^1^ Department of Neurointerventional, The First Affiliated Hospital of Jinzhou Medical University, Jinzhou, China; ^2^ Department of Acupuncture, Jin Zhou Hospital of Traditional Chinese Medicine, Jinzhou, China; ^3^ Department of Anesthesiology, The First Affiliated Hospital of Jinzhou Medical University, Jinzhou, China

**Keywords:** lung adenocarcinoma, brain, brain metastasis, pan-cancer analysis, genomic, genomic profiling, prognostic markers, immune infiltration

## Abstract

**Introduction:**

Lung adenocarcinoma (LUAD) has become one of the leading causes of cancer-related deaths globally, with metastasis representing the most lethal stage of the disease. Despite significant advances in diagnostic and therapeutic strategies for LUAD, the mechanisms enabling cancer cells to breach the blood-brain barrier remain poorly understood. While genomic profiling has shed light on the nature of primary tumors, the genetic drivers and clinical relevance of LUAD metastasis are still largely unexplored.

**Objectives:**

This study aims to investigate the genomic differences between brain-metastatic and non-brain-metastatic LUAD, identify potential prognostic biomarkers, and evaluate the efficacy of AH-6809 in modulating key molecular pathways involved in LUAD metastasis, with a focus on post-translational modifications (PTMs).

**Methods:**

Genomic analyses were performed using data from The Cancer Genome Atlas (TCGA) and the Gene Expression Omnibus (GEO). Differentially expressed genes (DEGs) between brain-metastatic and non-metastatic LUAD samples were identified. Key gene modules were determined using Weighted Gene Co-expression Network Analysis (WGCNA), and their prognostic significance was assessed through Kaplan-Meier analysis. Cellular experiments, including CCK8 and qRT-PCR assays, were conducted to evaluate the anti-cancer effects of AH-6809 in LUAD cells. Apoptosis and inflammatory marker expression were assessed using immunofluorescence.

**Results:**

Genomic analysis differentiated brain-metastatic from non-brain-metastatic LUAD and identified NLRP7, FIBCD1, and ELF5 as prognostic markers. AH-6809 significantly suppressed LUAD cell proliferation, promoted apoptosis, and modulated epithelial-mesenchymal transition (EMT) markers. These effects were reversed upon NLRP7 knockdown, highlighting its role in metastasis. Literature analysis further supported AH-6809’s tumor-suppressive activity, particularly in NLRP7 knockdown cells, where it inhibited cell growth and facilitated apoptosis. AH-6809 was also found to affect SUMO1-mediated PTMs and downregulate EMT markers, including VIM and CDH2. NLRP7 knockdown partially reversed these effects. Immunofluorescence revealed enhanced apoptosis and inflammation in lung cancer cells, especially in NLRP7 knockdown cells treated with AH-6809. The regulatory mechanisms involve SUMO1-mediated post-translational modifications and NQO1. Further studies are required to elucidate the molecular mechanisms and assess the clinical potential of these findings.

**Conclusion:**

These findings demonstrate the critical role of NLRP7 and associated genes in LUAD metastasis and suggest that AH-6809 holds promise as a potential therapeutic agent for brain-metastatic LUAD.

## Introduction

Lung adenocarcinoma (LUAD), the most common subtype of non-small cell lung cancer (NSCLC), is a leading cause of cancer-related mortality worldwide (Sainz De Aja et al., 2021; Ye et al., 2019). Despite advancements in diagnostic tools and therapeutic strategies, accurate prediction of LUAD progression, particularly metastasis, remains challenging. This is due to the reliance on indirect findings and a lack of understanding of the molecular mechanisms driving LUAD metastasis (Webb and Simon, 2010; Li et al., 2018). Brain metastasis occurs in approximately 20%–40% of patients with advanced LUAD, making it one of the most common sources of brain metastasis across cancer types (Yang et al., 2004; Valastyan and Weinberg, 2011). Metastasis, a complex multi-step process in which cancer cells spread from the primary tumor to distant organs, is regarded as the most lethal phase of cancer progression (Shi et al., 2016; Kleczko et al., 2019). LUAD cells enhance their invasive capabilities and promote metastasis, including to the brain, through epithelial-mesenchymal transition (EMT). These cells also stimulate angiogenesis, alter the local brain microenvironment, and create favorable conditions for metastatic growth. Additionally, LUAD cells evade immune responses, further complicating the treatment of metastasis (Chen et al., 2020; Li et al., 2021).

While genomic profiling has provided significant insights into the genetic and molecular drivers of primary LUAD tumors, particularly through efforts like The Cancer Genome Atlas (TCGA) project, the molecular mechanisms specific to LUAD brain metastasis remain largely unexplored. This gap includes key gene alterations, such as EGFR mutations and ALK rearrangements, which are associated with a higher risk of brain metastasis (Li et al., 2018; Xie et al., 2021). Much of the work in cancer research focuses on genomics, which calls for cross-cancer analyses to study pan-cancers like LUAD together with other types both genetic and molecular basis providing global understanding around diseases (Liu and Zhang, 2014; Shi et al., 2016). While the existing literature has identified various genes and epigenetic changes associated with LUAD, the heterogeneity and dynamism of metastatic disease present significant challenges in identifying consistent biomarkers and therapeutic targets (Mastrogiacomo et al., 2024; Testa et al., 2022). Previous genomic studies have predominantly focused on primary tumors, with limited attempts to link genomic alterations to metastatic potential (Mardis, 2018).

In the existing LUAD studies, many genes have been found associated with primary tumors, but their direct relationship with metastatic potential remains unclear (Minn et al., 2005; Yao et al., 2020). Although the roles of genes like ARRDC5 and ELF5 in cancer progression have been studied, their pan-cancer significance across different cancer types has not been fully demonstrated (Mishra et al., 2016; Piggin et al., 2016). Additionally, the downregulation of L1CAM in LUAD metastasis contrasts with its known role in promoting invasion in other cancers, revealing a more complex and context-dependent role of L1CAM in cancer biology (Wu et al., 2019; Hai et al., 2012). Current research on these genes highlights the need for a deeper investigation into their prognostic potential and their roles in mediating the metastatic cascade in LUAD. Furthermore, while some therapeutic strategies have targeted these pathways (Guo et al., 2022; Yamaguchi et al., 2024), their efficacy in treating brain-metastatic LUAD is still under investigation. Identifying molecular markers that can predict metastasis and guide therapy is critical to improve patient outcomes. Though an overall understanding of the genomic signature has been established through studies on LUAD, metastatic disease is rarely studied and done so with a small sample size (Li et al., 2021; Feng et al., 2022). In addition, Conducting such analysis in existing studies that are mainly based on public genomic data (having advantages of large scope but maybe lack detailed information) can be challenging (Näpflin et al., 2019; Liang and Greenwood, 2015). Further studies are required to verify these genes and their functional roles in LUAD metastasis, using experimental methods (Wang et al., 2020; Zhang et al., 2021).

The integration of big data and bioinformatics has become increasingly important for identifying biomarkers that can aid in the diagnosis and prognosis of LUAD (Takano and Ito, 2023; Yuan et al., 2024). Protein-protein interaction networks, as well as their modifications and regulatory mechanisms, are central to understanding cellular signaling and functional regulation (Wu et al., 2024; Tian et al., 2023). In the field of genomics, unsurpassed work in immune microenvironment-related diagnosis and prognostic evaluation for many diseases has been achieved through transcriptome (Cao et al., 2024; Zhao et al., 2024). Advances in transcriptomic analysis have also provided valuable insights into the immune microenvironment and its role in disease progression (Thakur et al., 2023; Wu et al., 2023). By employing cutting-edge methodologies such as machine learning, multi-omics analysis, high-throughput sequencing, and bioinformatics, researchers continue to explore new therapeutic strategies that open doors to precision medicine and personalized treatments (Wu et al., 2023; Zhuo et al., 2024; Aydoğdu et al., 2024).

This study aims to address these gaps by conducting a comprehensive genomic analysis of LUAD metastasis. Previous research has identified various genetic alterations in LUAD, but few have linked these changes specifically to brain metastasis. The clinical implications of these genes remain largely unexplored, and while certain therapeutic agents targeting these pathways show promise, their efficacy in treating brain-metastatic LUAD is still being evaluated. Further studies are necessary to determine the molecular mechanisms behind these observations and assess their potential for clinical application. This version streamlines the background, enhances clarity, and aligns the content with the formal tone typical of SCI papers, making it concise yet comprehensive for a scientific audience.

## Materials and methods

### Acquisition and processing of GEO datasets

In this study, microarray data from brain metastasis samples of lung adenocarcinoma patients were analyzed. A total of 28 samples were included, with 19 obtained from Marc Ladanyi’s research group and nine from William L. Gerald’s research group. The data were retrieved from the Gene Expression Omnibus (GEO) database provided by the National Center for Biotechnology Information (NCBI) under the accession number GSE14108. Additionally, to investigate the tumor microenvironment in brain metastases of lung adenocarcinoma patients, bulk RNA sequencing was performed on brain metastasis samples from six patients, utilizing the Illumina HiSeq X Ten platform. These sequencing data were also acquired from the GEO database, with accession number GSE141685. Differential expression analysis was conducted by comparing the expression profiles of brain metastasis samples to those of primary lung adenocarcinoma tumors obtained from the TCGA database. Specifically, data from 14 early-stage and 11 late-stage primary lung adenocarcinoma tumors were included in the analysis. Statistical analysis was performed using the False Discovery Rate (FDR) for multiple testing correction with a significance threshold set at *p* < 0.05. Data normalization was applied, and the linear model coupled with empirical Bayes methods from the R package “limma” was employed to identify significantly differentially expressed genes.

### Pan-cancer expression analysis of core genes

This study encompassed the mRNA expression data, copy number variation (CNV) data, and DNA methylation 450 K data from 20 cancer types, including bladder urothelial carcinoma (BLCA), breast invasive carcinoma (BRCA), cholangiocarcinoma (CHOL), colon adenocarcinoma (COAD), cervical and endocervical cancers (CESC), esophageal carcinoma (ESCA), glioblastoma multiforme (GBM), head and neck squamous cell carcinoma (HNSC), kidney chromophobe (KICH), kidney renal clear cell carcinoma (KIRC), kidney renal papillary cell carcinoma (KIRP), liver hepatocellular carcinoma (LIHC), lung adenocarcinoma (LUAD), lung squamous cell carcinoma (LUSC), pancreatic adenocarcinoma (PAAD), prostate adenocarcinoma (PRAD), rectum adenocarcinoma (READ), stomach adenocarcinoma (STAD), thyroid carcinoma (THCA), and uterine corpus endometrial carcinoma (UCEC). These data, which included both tumor and normal samples, were downloaded from Firehose (http://gdac.broadinstitute.org). Mutation, miRNA sequencing data, and clinical information were obtained from the Xena Browser (https://xenabrowser.net/datapages/). To assess the differences in gene expression between cancerous and normal tissues across various cancer types, we utilized the Wilcoxon rank-sum test (also known as the Mann-Whitney U test), a non-parametric method suitable for comparing the medians of two independent groups without requiring assumptions of normality. Statistical significance was determined with an alpha value of 0.05. TPM expression data for tumor samples from TCGA and normal samples from GTEx were accessed via the UCSC Xena database. Z-score standardization was applied to normalize expression data and mitigate dimensional discrepancies across datasets. To enhance the evaluation of gene copy number alterations, both heterozygous and homozygous amplifications and deletions were considered. Pearson’s correlation coefficients were calculated between gene expression levels and copy number segment values to assess the relationship between CNV and gene expression.

### Core gene promoter methylation analysis

We conducted an in-depth methylation analysis of several key genomic regions, including the TSS1500 region (1,500 base pairs upstream of the transcription start site), the TSS200 region (within 200 base pairs of the transcription start site), the first exon, and the 5'untranslated region (5'UTR). Methylation values across these regions were then tallied and the median methylation value was calculated to provide a global index of DNA methylation status for each sample. To explore potential correlations between methylation levels and gene expression, we used Spearman’s rank correlation, a non-parametric method for assessing the monotonic relationship between two variables that do not follow a normal distribution. The methylation levels were defined as the independent variable, and gene expression levels were being dependent on these exposure of an exogenous linear regression was pursued to calculate their associations by computing Spearman rank correlation coefficient. Further, promoter region methylation was compared in tumors and normal samples using Wilcoxon rank-sum test. This method is able to identify large changes in methylation levels between tumor and normal tissues.

### Analysis of core genes using ATAC-seq

In this study, we utilized the ChIPseeker package to analyze and visualize ATAC-seq data. Using its annotatePeak function allowed us to closely examine transcription start sites within gene promoter areas, setting the tssRegion parameter to c (-3,000, 3,000) to ensure coverage from 3,000 base pairs upstream to downstream of the transcription start site. This thorough investigation around the transcription start site is critical for comprehending the scattering of transcription factor binding locations and histone changes. Additionally, the covplot function created coverage plots visually portraying the dispersion of peaks across the genome from our ATAC-seq data. The plots supplied specific information too, like gene names, cancer types, chromosomal positions, and precise genetic distances. These comprehensive genomic visualization tools provide researchers profound insights into the genomic landscape, with some plots showing broad trends of open chromatin regions across whole chromosomes and others zooming in to examine the distribution of TF binding or histone marks around individual gene promoters.

### GSEA enrichment analysis in pan-cancer studies

It searched the TCGA database and collected RNA-seq or microarray data for various types of cancer, that included both expression information on tumor and normal (or paired) samples. These outcome data were put through rigorous quality control before analysis to guarantee sample and probe precision. The data would then be standardized to reduce technical variability. Differential expression analysis was carried out using the “R package ‘clusterProfiler’” “limma”, a tool for normalization of data, background correction and statistical testing to find significantly differentially expressed genes. Genes were screened based on a log2 fold change (log_2_FC) and *p*-value, with log_2_FC representing the logarithmic ratio of gene expression changes and the *p*-value indicating statistical significance. Next, we used the R package “clusterProfiler” in pathway enrichment analysis of differentially expressed genes against databases such as KEGG, GO and Reactome by gene set enrichment analysis (GSEA). The enrichment score (ES), ranging from 0 to 1, was used to assess the correlation between biological processes and gene expression changes. Finally, the R package “ggplot2″ was used to visualise data more conveniently with intuitive graphs and charts such as bar plots, scatter plots and gene expression heatmaps. This analytical framework provides a systematic approach to understanding the molecular mechanisms underlying cancer gene expression.

### Molecular characterization of core genes in LUAD

We conducted a ROC curve analysis using the pROC package to evaluate the diagnostic performance of the single-sample gene set enrichment analysis score (ssGSEAscore) in distinguishing LUAD from normal control groups. This evaluation involved calculating the 95% confidence interval---the area under the curve (AUC) and plotting the smoothed ROC curve. The ssGSEAscore was derived from the TCGA dataset’s RNA-seq data using the “ssgsea” method of the GSVA package. Data was sourced from the PanCanAtlas, specifically the EBPlusPlusAdjustPANCAN_IlluminaHiSeq_RNASeqV2 geneExp.tsv file processed by the Firehose pipeline with MapSplice + RSEM and normalized by setting the upper quartile to 1,000. Further analysis involved using the Wilcoxon rank-sum test to compare ssGSEAscore expression levels between tumor and normal tissues within the LUAD dataset, assessing their statistical significance. The Wilcoxon signed-rank test also compared expression differences between tumor and adjacent non-tumor tissues. Calibration curves described the consistency between model predictions and actual observations, while goodness-of-fit tests evaluated the model’s alignment with ideal conditions.

The Wilcoxon rank-sum test was also used to analyze differences in ssGSEAscore distributions between early- and late-stage LUAD samples. A Kruskal-Wallis rank-sum test then assessed further whether this was merely due to chance within the LUAD data set. These methods represent robust statistical tools for evaluating how gene set expression profiles correspond with clinical characteristics in lung adenocarcinoma.

### Survival prognosis analysis of core genes in LUAD

Kaplan-Meier survival analysis used the survival package in R environment determined optimal high and low ssGSEAscore group cut-off values. The survminer package ensured each score group had a minimum 0.3 proportion. The log-rank test assessed survival differences between the two groups using the survfit function. Additionally, univariate Cox survival analysis results were combined using the inverse variance method in a meta-analysis, with hazard ratio serving as the primary measurement.

### Connectivity map (cMAP) analysis

To identify potential therapeutic options that could mitigate the tumor-promoting effects driven by specific genes, a Connectivity Map (cMAP) analysis was performed using CMAP_gene_signatures. The associated RData file comprises gene expression profiles for 1,288 different compounds. A gene signature was generated by selecting the 150 most significantly upregulated and 150 most significantly downregulated genes, based on a comparison between patients with high and low levels of gene expression in tumors. The eXtreme Sum (XSum) algorithm was employed to compare these gene signatures with cMAP signatures, generating similarity scores for the 1,288 compounds. The analysis followed established protocols as described in prior studies (Yang et al., 2022; Malta et al., 2018).

### Cell lines and clinical samples

The non-small cell lung cancer (NSCLC) cell line, NCI-H1299 (ATCC^®^ CRL-5803™), was obtained from the American Type Culture Collection (ATCC, United States). These cells were cultured in Dulbecco’s Modified Eagle’s Medium (DMEM), supplemented with 10% fetal bovine serum (FBS).

### Silencing of NLRP7 gene using lentiviral vectors

To assess the impact of NLRP7 gene knockdown, lentiviral vectors were employed to silence the gene in NLRP7 cells. The specific targeting sequences were sourced from Open Biosystems.

### Immunofluorescence

Cells were seeded into 24-well culture plates and incubated overnight to allow for attachment. Fixation was performed at room temperature using 3.7% paraformaldehyde for 15 min, followed by permeabilization in chilled methanol at −20°C for another 15 min. Afterward, cells were incubated at room temperature for 1 h in blocking buffer (PBS containing 5% normal goat serum and 0.5% Triton X-100). Primary antibodies were added, and the cells were incubated overnight at 4°C. The next day, cells were washed three times with PBS, 10 min each time, and then incubated at room temperature with a goat anti-rabbit secondary antibody conjugated to a fluorophore (diluted 1:500 in blocking buffer). Prior to imaging, the nuclei were stained at room temperature with DAPI for 30 min (catalog number D9542; Sigma). Images were captured using a Nikon Eclipse E800 fluorescence microscope.

### Quantitative real-time PCR (qRT-PCR)

Total RNA was isolated with TRIzol, and complementary DNA (cDNA) synthesis was carried out using the FastStart Universal SYBR Green Master (ROX) from Roche, Switzerland, in conjunction with the PrimeScript™ RT Reagent Kit from TaKaRa, Japan. The qRT-PCR was conducted using a CFX96™ Real-Time System combined with a C1000™ Thermal Cycler, both from Bio-Rad, United States.

### CCK-8 assay

The CCK-8 assay was utilized to evaluate cell proliferation. In brief, 2000 cells were seeded into each well of a 96-well plate and incubated at 37°C under 5% CO_2_. Afterward, 10 µL of CCK-8 solution (Vazyme Biotech Co., Ltd.) was added to each well, and the plate was incubated for an additional 2 h at 37°C. As a result, the absorbance of 450 nm was determined by microplate reader assay. Cell proliferation curves were constructed based on the three independent experiments.

### Plate colony formation assay

A plate colony formation assay was employed to evaluate the long-term proliferative capacity of lung adenocarcinoma cells, Briefly, LUAD cells were transfected with shRNA targeting NLRP7, or cells were treated with AH-6809, to evaluate their effect on the clonogenic prospective. Cells were plated into six-well dishes in a density of 500 cells per well, incubated at 37°C under 5% CO₂ and kept in a humid environment. The culture medium was changed every 3 days to maintain optimal growth conditions After visible colonies had appeared (10–14 days after plating), the cells were fixed with 4% paraformaldehyde at room temperature for 15 min. Following fixation, the colonies were stained with 0.1% crystal violet solution for 30 min, and then gently washed with phosphate-buffered saline (PBS) and air-dried.

The number of visible colonies containing more than 50 cells was then counted under a microscope, and the colony formation efficiency-ratio of number colonies to cells originally plated (as a percentage) was calculated. This assay was performed in triplicate, and the results were analyzed statistically using the Student’s t-test to evaluate the significance of differences between experimental groups.

### Statistical analyses

Data were expressed as mean ± standard deviation (SD) and were processed using GraphPad Prism version 8. For comparing two groups, a t-test was employed, while a one-way analysis of variance (ANOVA) was utilized for comparisons across multiple groups. Correlation analysis was performed using the Pearson correlation coefficient. A *p*-value of less than 0.05 was regarded as indicative of statistical significance.

## Results

### Differentially expressed gene analysis of brain metastasis in lung adenocarcinoma

**FIGURE 1:**
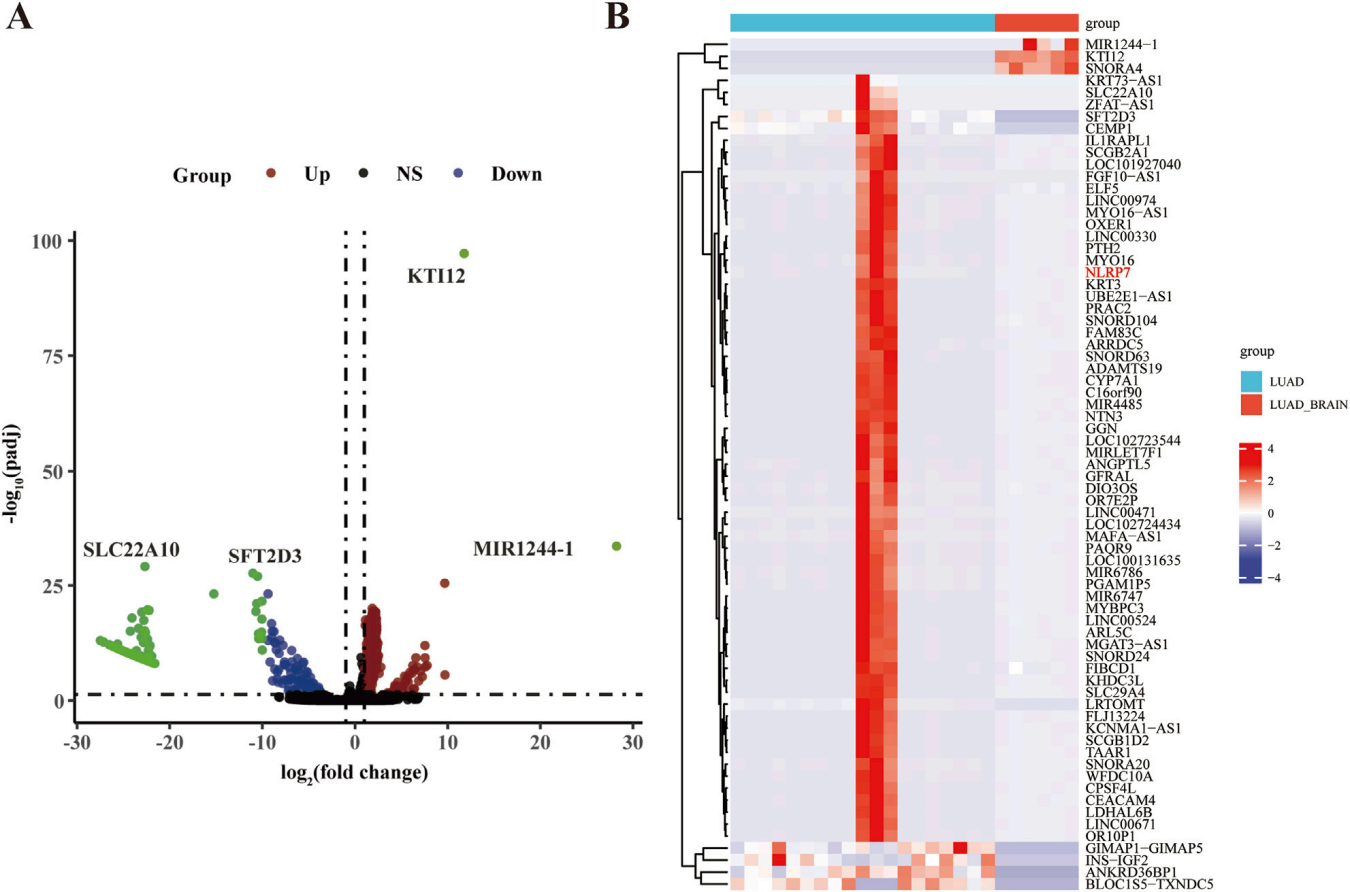
Analysis of GEO dataset identifying genes associated with brain metastasis in lung cancer. **(A)** Volcano plot depicting differentially expressed genes between lung adenocarcinoma (LUAD) and brain metastases of LUAD (LUAD_brain). The x-axis represents the log^2^ fold change, and the y-axis represents the -log10 *p*-value. Genes significantly upregulated in brain metastases are indicated in red, downregulated genes in green, non-significant (NS) genes in black, and genes with no significant change (NS) in blue. Notable genes such as SLC7A10, SFT2D3, KTI12, and MIR1244-1 are highlighted. **(B)** Heatmap showing the expression profiles of the top differentially expressed genes in LUAD and LUAD_brain samples. The expression values are normalized and represented as a color gradient, with red indicating higher and blue indicating lower expression. The hierarchical clustering on the left group genes is based on their expression patterns across samples. The groups (LUAD and LUAD_brain) are labeled at the top, with LUAD in red and LUAD_brain in cyan.

Through analysis of the GEO dataset, our comprehensive study focused on identifying genes associated with brain metastasis in LUAD. The analysis revealed significantly altered gene expression patterns between primary LUAD tumors and brain metastasis samples. As shown in Supplementary Figure S1A, a standardized boxplot compares the expression values between primary LUAD tumors and brain metastasis samples, illustrating the differences in gene expression between these tissue types. The distribution of gene expression highlighted significant differences between primary and metastatic tissues, with brain metastasis samples showing distinct patterns of gene regulation. To further explore the separation between primary and metastatic samples, we performed principal component analysis (PCA) (Supplementary Figure S1B). Additionally, the volcano plot highlights the differential expression of key genes between LUAD and LUAD brain metastasis samples. The plot shows genes significantly upregulated in brain metastasis, such as SLC7A10, SFT2D3, KTI12, and MIR1244-1, and genes significantly downregulated (Figure 1A). The heatmap presents normalized expression values on a color scale (Figure 1B). Clustering based on gene expression profiles reveals a clear separation between primary LUAD tumor samples and LUAD brain metastasis samples (in cyan). These findings identify potential therapeutic targets for future research and emphasize the importance of understanding the molecular basis of brain metastasis in LUAD.

### Enrichment analysis of genes associated with lung cancer brain metastasis

**FIGURE 2:**
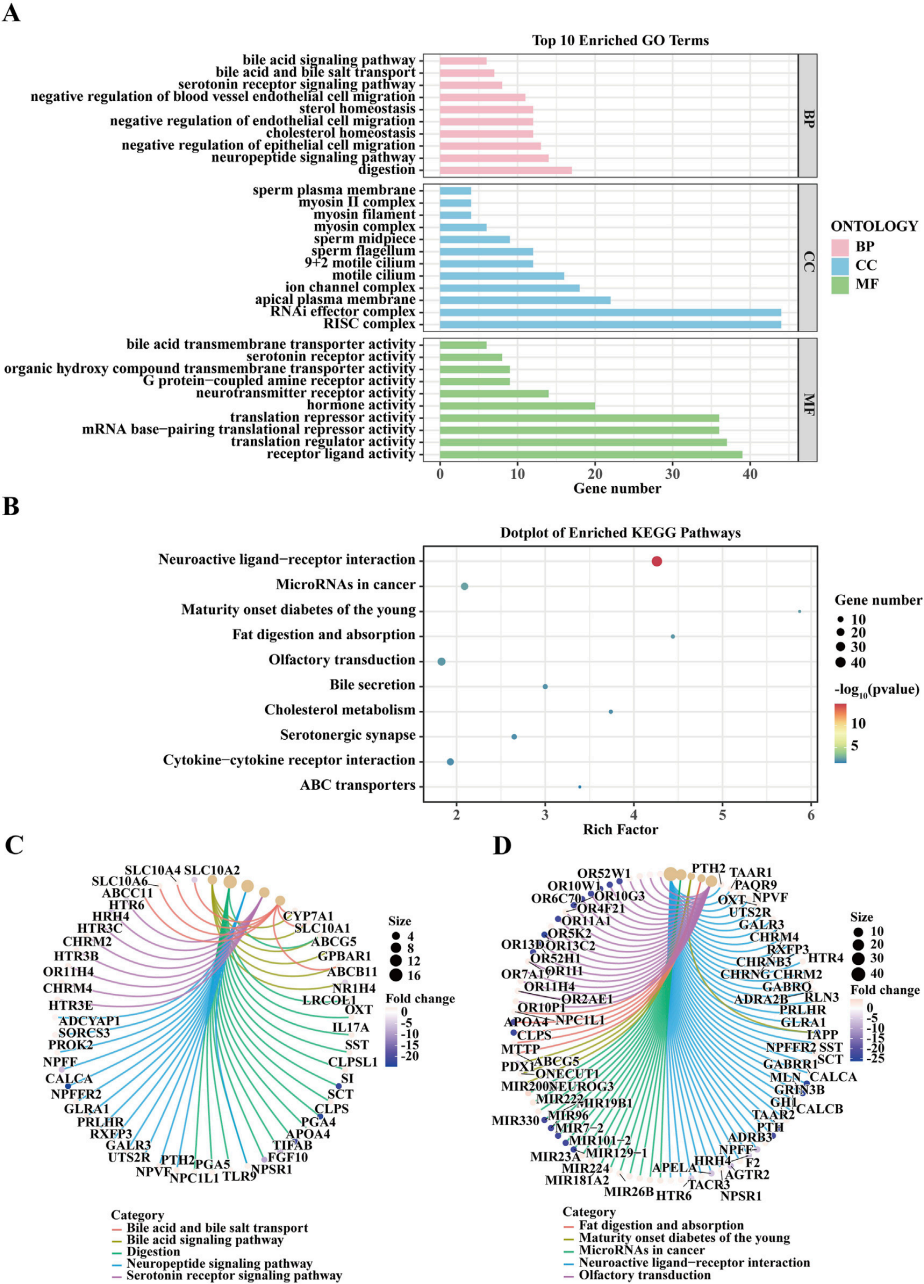
Enrichment Analysis of Genes Associated with Lung Cancer Brain Metastasis from GEO Dataset. **(A)** The bar plot displays the top 10 Gene Ontology (GO) terms enriched in genes associated with lung cancer brain metastasis. GO terms are categorized into three ontologies: Biological Process (BP), Cellular Component (CC), and Molecular Function (MF). The x-axis represents the gene count, while the y-axis lists the enriched GO terms. Significant GO terms include bile acid biosynthetic process, organic hydroxy compound metabolic process, and sperm plasma membrane (BP); mitochondrion, and synaptic membrane (CC); and bile acid transmembrane transporter activity, organic hydroxy compound transporter activity, and mRNA 3'-end processing (MF). The enrichment analysis was performed using the GEO dataset, and significance was determined with adjusted *p*-values. **(B)** The dot plot illustrates the enriched Kyoto Encyclopedia of Genes and Genomes (KEGG) pathways for genes related to lung cancer brain metastasis. The x-axis shows the rich factor, and the y-axis lists the KEGG pathways. Dot size represents the number of genes, and color indicates the *p*-value. Notable pathways include Neuroactive ligand-receptor interaction, MicroRNAs in cancer, Fat digestion and absorption, and Olfactory transduction. **(C)** The chord diagram visualizes the relationship between enriched GO terms and their associated genes. Each GO term is connected to the corresponding genes, highlighting the shared and unique associations among the terms. This representation provides an overview of the functional connections and biological processes involved in lung cancer brain metastasis. **(D)** This chord diagram shows the relationships between enriched KEGG pathways and their associated genes. Similar to **(C)**, the connections illustrate the shared and unique gene associations among different pathways, revealing the intricate network of biological interactions relevant to lung cancer brain metastasis.

In this study, we performed an enrichment analysis of genes associated with lung cancer brain metastasis using data from the GEO dataset. The top 10 enriched GO terms, categorized into Biological Process (BP), Cellular Component (CC), and Molecular Function (MF), indicate key biological activities such as bile acid biosynthetic process, organic hydroxy compound metabolic process, and sperm plasma membrane (BP); mitochondrion and synaptic membrane (CC); and bile acid transmembrane transporter activity, organic hydroxy compound transporter activity, and mRNA 3'-end processing (MF) (Figure 2A). The enriched KEGG pathways, including neuroactive ligand-receptor interaction, microRNAs in cancer, fat digestion and absorption, and olfactory transduction (Figure 2B). The chord diagrams in Figures 2C, D visualize the relationships between enriched GO terms and KEGG pathways alongside their associated genes, highlighting both shared and unique gene associations, thereby offering a comprehensive overview of the functional connections and biological processes relevant to lung cancer brain metastasis. These findings emphasize critical biological processes, cellular components, molecular functions, and pathways potentially implicated in the pathogenesis and progression of lung cancer brain metastasis, providing valuable insights for future research and potential therapeutic targets.

### Analysis reveals five L1CAM-Related prognostic genes in lung cancer brain metastasis

**FIGURE 3:**
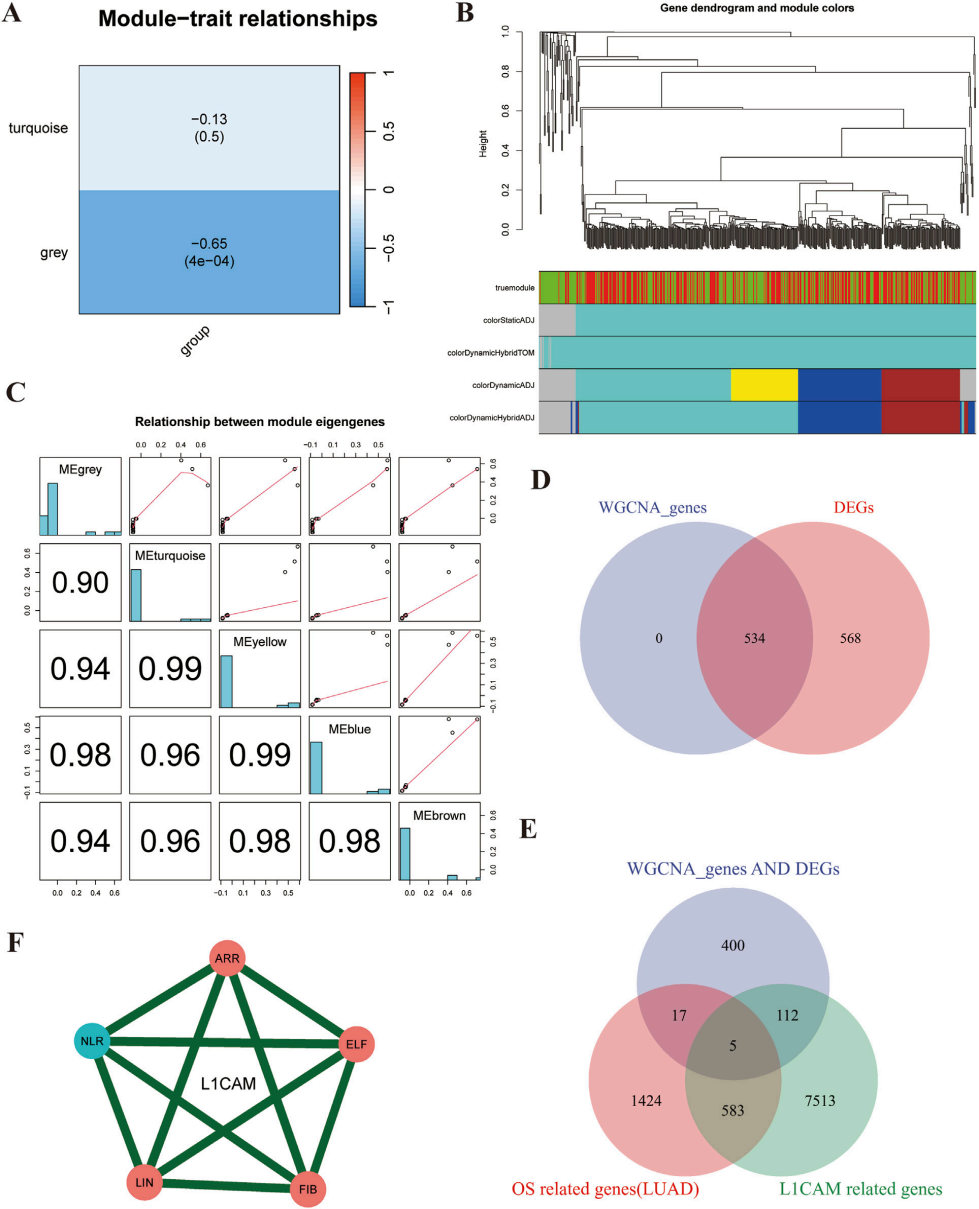
WGCNA analysis reveals five L1CAM-related prognostic genes in lung cancer brain metastasis. **(A)** Heatmap showing the correlation between identified modules and the trait of interest (group). The turquoise module has a weak negative correlation (−0.13, *p* = 0.5), while the grey module shows a stronger negative correlation (−0.65, *p* < 0.001), indicating a significant association. **(B)** Hierarchical clustering dendrogram of genes, with different colors representing distinct modules identified by WGCNA. Modules correspond to highly interconnected gene clusters. **(C)** Scatter plots and correlation matrices showing the relationships between module eigengenes. High correlation values (up to 0.99) indicate significant co-expression among genes within these modules. **(D)** Comparison of genes identified by WGCNA (left circle) and differentially expressed genes (DEGs) (right circle). The intersection reveals 534 overlapping genes, suggesting these are critical in the trait of interest. **(E)** Venn Diagram of WGCNA Genes, DEGs, and L1CAM-Related Genes: Overlap of WGCNA genes (blue), DEGs (pink), and L1CAM-related genes (green). The intersection indicates five common genes (highlighted in the center), likely key prognostic markers for lung cancer brain metastasis. **(F)** Interaction network of the five identified L1CAM-related prognostic genes (L1CAM, ARR, NLR, ELF, LIN, and FIB). The network shows strong interactions (green lines) among these genes, suggesting their cooperative role in lung cancer brain metastasis.

In this study, we used Weighted Gene Co-Expression Network Analysis (WGCNA) to identify prognostic genes associated with L1CAM in lung cancer brain metastasis. The hierarchical clustering dendrogram of LUAD and brain metastasis samples was used to detect outliers and visualize sample clustering (Supplementary Figure S1C). The scale independence and mean connectivity plots confirmed the scale-free topology of the co-expression network (Supplementary Figure S1D). The node connectivity distribution indicated that certain highly connected hubs play central roles in the network (Supplementary Figure S1E). The module eigengene clustering dendrogram revealed co-expression modules composed of highly correlated genes, offering insights into network structure in LUAD and brain metastasis samples (Supplementary Figure S1F). Module-trait correlations (Figure 3A) showed a weak negative correlation in the turquoise module and a stronger negative correlation in the grey module. The gene dendrogram and module colors are shown in Figure 3B. Correlation matrices and scatter plots demonstrated strong correlations (up to 0.99) between module eigengenes (Figure 3C). A Venn diagram (Figure 3D) revealed 534 overlapping genes between WGCNA-identified genes and differentially expressed genes (DEGs). Further analysis identified five key L1CAM-related genes (L1CAM, ARRDC5, NLRP7, ELF5, LINC00494, FIBCD1) (Figure 3E). Finally, network analysis of these genes (Figure 3F) illustrated strong interactions, suggesting their cooperative role in lung cancer brain metastasis.

### Pan-cancer expression landscape of core genes

**FIGURE 4:**
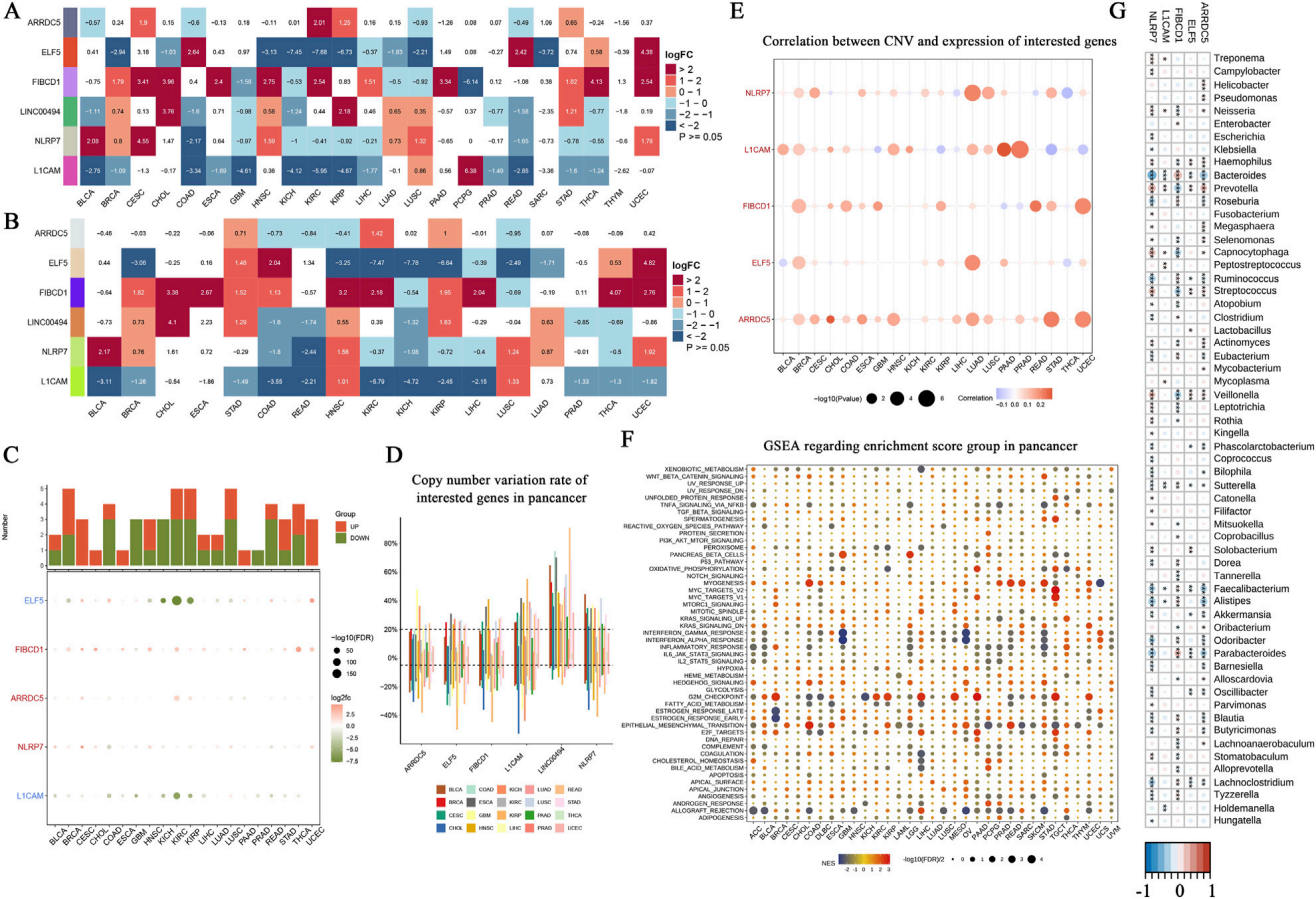
Pan-cancer landscape analysis of core gene expression, copy number variation, and functional enrichment. **(A)** Heatmap showing the differential expression analysis of core genes (ARRDC5, ELF5, FIBCD1, LINC00494, NLRP7, L1CAM) across various cancer types (unpaired samples). The log fold change (logFC) values represent the expression differences between tumor samples and normal tissues, with red indicating upregulation and blue indicating downregulation. Statistical significance is denoted by *p* < 0.05. **(B)** Heatmap representing differential expression of the same core genes, but comparing paired cancer and adjacent normal tissue samples. The paired analysis aims to reduce inter-patient variability, with logFC values following the same color scheme as in **(A)**. **(C)** Bar chart and dot plot summarizing the differential expression analysis using TCGA-GTEx datasets. The bar chart at the top illustrates the number of cancer types where each gene is significantly upregulated (red) or downregulated (blue). The dot plot below indicates the logFC for each cancer type, with the dot size corresponding to the statistical significance (-log10(FDR)). **(D)** Copy number variation (CNV) rate of the core genes across different cancer types. The boxplot shows the variation in CNV rates, with cancer types represented by different colors, highlighting genes with significant copy number alterations across the pan-cancer dataset. **(E)** Correlation analysis between CNV and expression of the core genes. The bubble plot demonstrates the relationship between CNV and gene expression levels in multiple cancer types, with the size of the bubbles representing the strength of the correlation. **(F)** Gene Set Enrichment Analysis (GSEA) results for the core genes across different cancer types. The dot plot shows the enriched pathways, with the size of the dots reflecting the significance of enrichment (-log10(*p*-value)) and color indicating the direction of enrichment (red for positive enrichment, blue for negative enrichment). **(G)** Tumor microbiome analysis across various cancer types. The heatmap illustrates the relative abundance of different microbial species identified in tumor samples, providing insights into the potential role of the tumor microbiome in cancer progression and gene expression.

In this study, we examined the expression patterns of seven core genes (ARRDC5, ELF5, FIBCD1, LINC00494, NLRP7, L1CAM) across multiple cancer types using a combination of unpaired and paired samples, along with data from the TCGA-GTEx datasets. A heatmap of the differential expression analysis for these core genes across various cancer types using unpaired samples is shown (Figure 4A). This analysis revealed significant variations in gene expression across different cancer types. For example, L1CAM exhibited significant overexpression in LUSC, whereas ARRDC5 showed marked upregulation in BRCA, suggesting the involvement of these genes in tumorigenesis. The paired sample analysis further refined these findings by comparing cancer and adjacent normal tissues, minimizing inter-patient variability. Significant downregulation of ELF5 was observed in paired COAD samples, suggesting its potential role as a tumor suppressor in this cancer type (Figure 4B). Other genes, such as NLRP7 and ARRDC5, also displayed consistent dysregulation across paired samples, reinforcing their potential as therapeutic targets. Next, the TCGA-GTEx datasets were used to provide a broader view of gene expression across various cancer types and conditions. A bar chart and dot plot summarize the number of cancer types where each gene is significantly upregulated or downregulated (Figure 4C). This comprehensive analysis emphasized ARRDC5 and NLRP7 as the most consistently dysregulated genes across multiple cancer types, underscoring their critical role in cancer biology. Copy number variation (CNV) analysis revealed notable alterations in several of the core genes across different cancer types. For instance, ARRDC5 and L1CAM demonstrated significant CNV alterations, suggesting that these genetic changes may contribute to their differential expression and oncogenic potential (Figure 4D). The correlation between CNV and gene expression was further explored, with a bubble plot illustrating the correlation strength. Larger bubbles indicate a stronger correlation, with ARRDC5 demonstrating a positive correlation between CNV and expression in breast invasive carcinoma (Figure 4E). This result reinforces the hypothesis that CNV plays a role in regulating gene expression in cancer. To explore the functional implications of these findings, we performed a GSEA to identify the biological pathways associated with the dysregulated genes. Key pathways, such as apoptosis, immune response, and cell cycle regulation, were enriched for these genes (Figure 4F). For instance, NLRP7 was particularly associated with immune response pathways, indicating its involvement in modulating the tumor immune microenvironment. Finally, tumor microbiome analysis across various cancer types identified possible interactions among microbial species and gene expression. The heatmap shows the relative abundance of microbial species in tumor samples, with certain species more prevalent in lung and gastrointestinal cancers, potentially impacting the gene expression profiles observed in these tumors (Figure 4G).

### Core gene promoter methylation analysis and its correlation with gene expression

The comprehensive relationship between gene promoter methylation in the core promoter and gene expression levels is displayed in Supplementary Figure S1G, H. This analysis captured the variance between LUAD and brain metastasis samples, confirming clear clustering and separation between the two groups. The heatmap analysis of promoter methylation levels for the core genes across the samples revealed significant variations in methylation, as shown in Supplementary Figure S1G. Blue cells indicate lower methylation levels, while red cells denote higher methylation levels, illustrating the wide range of epigenetic modifications across different patient samples. This trend is particularly prominent in ARRDC5 and NLRP7, which exhibit significant methylation variability between LUAD and brain metastasis tissues. Regarding the correlation between gene expression and promoter methylation, Supplementary Figure S1H shows the relationship between mRNA expression levels and promoter methylation across the same core genes.

### Promoter methylation analysis of core genes

The study analyzed the promoter regions of six core genes and their methylation patterns. Methylation levels and distributions were scrutinized; samples were classified by methylation status; methylation peaks’ concentration and its relationship with various genomic characteristics were recorded. Promoter Methylation of ARRDC5 (Supplementary 60% of the promoter showed hypermethylation and 20% showed hypomethylation. (Figure 2A). Consensus Coding Sequence revealed that mostly methylation occurred in exonic areas or in promoter regions; however, at promoter level there were discrete peaks which were usually concentrated. ELF5 Promoter Methylation Analysis (Supplementary Figure S2B) showed a balanced distribution of methylation with significant peaks in CpG islands and promoter regions. FIBCD1 Promoter Methylation Analysis (Supplementary Figure S2C) indicated high methylation levels, with 70% hypermethylation and concentrated peaks within the promoter region. L1CAM Promoter Methylation Analysis (Supplementary Figure S2D) presented moderate methylation with 60% hypermethylation and significant peaks in CpG islands and promoter regions. LINC00494 Promoter Methylation Analysis (Supplementary Figure S2E) exhibited high hypermethylation (80%), with dense methylation in gene bodies and promoter regions. NLRP7 Promoter Methylation Analysis (Supplementary Figure S2F) revealed predominant hypermethylation (90%) with prominent peaks within the promoter region. Overall, these findings indicate distinct methylation patterns in the promoter regions of these core genes, suggesting significant variability and a common feature of hypermethylation, which could play a crucial role in regulating gene expression.

### Correlation of core gene expression with LUAD prognosis

The study investigated the relationship between ssGSEAscore expression and LUAD prognosis. The calibration curve and goodness-of-fit test for ssGSEAscore expression in predicting tumor versus normal groups showed a satisfactory model fit, as indicated by the Hosmer-Lemeshow test (*p* = 0.555) (Supplementary Figure S3A). The differential expression analysis revealed significantly higher ssGSEAscore in LUAD tumor groups compared to normal groups (Supplementary Figure S3B). However, paired difference analysis between normal and tumor tissues showed no significant difference (*p* = 0.559) (Supplementary Figure S3C). Further analysis depicted the ssGSEAscore expression across the four clinical stages of LUAD. The violin plots indicated variability in ssGSEAscore with disease progression from Stage I to Stage IV (Supplementary Figure S3D). A comparison between early-stage (Stage I-II) and late-stage (Stage III-IV) LUAD demonstrated significant differences in ssGSEAscore expression, suggesting its potential role in disease progression (Supplementary Figure S3E). Median ssGSEAscore values across the four stages, along with a line plot overlay, indicated the trend of expression with advancing disease (Supplementary Figure S3F). Finally, the diagnostic efficacy of ssGSEAscore in distinguishing tumors from normal tissues in LUAD patients was evaluated using a ROC curve. The AUC was 0.574, with a 95% confidence interval of 0.506–0.640, indicating a moderate diagnostic ability (Supplementary Figure S3G). These results highlight the potential utility of ssGSEAscore as a biomarker for LUAD prognosis and underscore the variability in its expression with disease progression.

### Core gene survival prognosis analysis in LUAD

**FIGURE 5:**
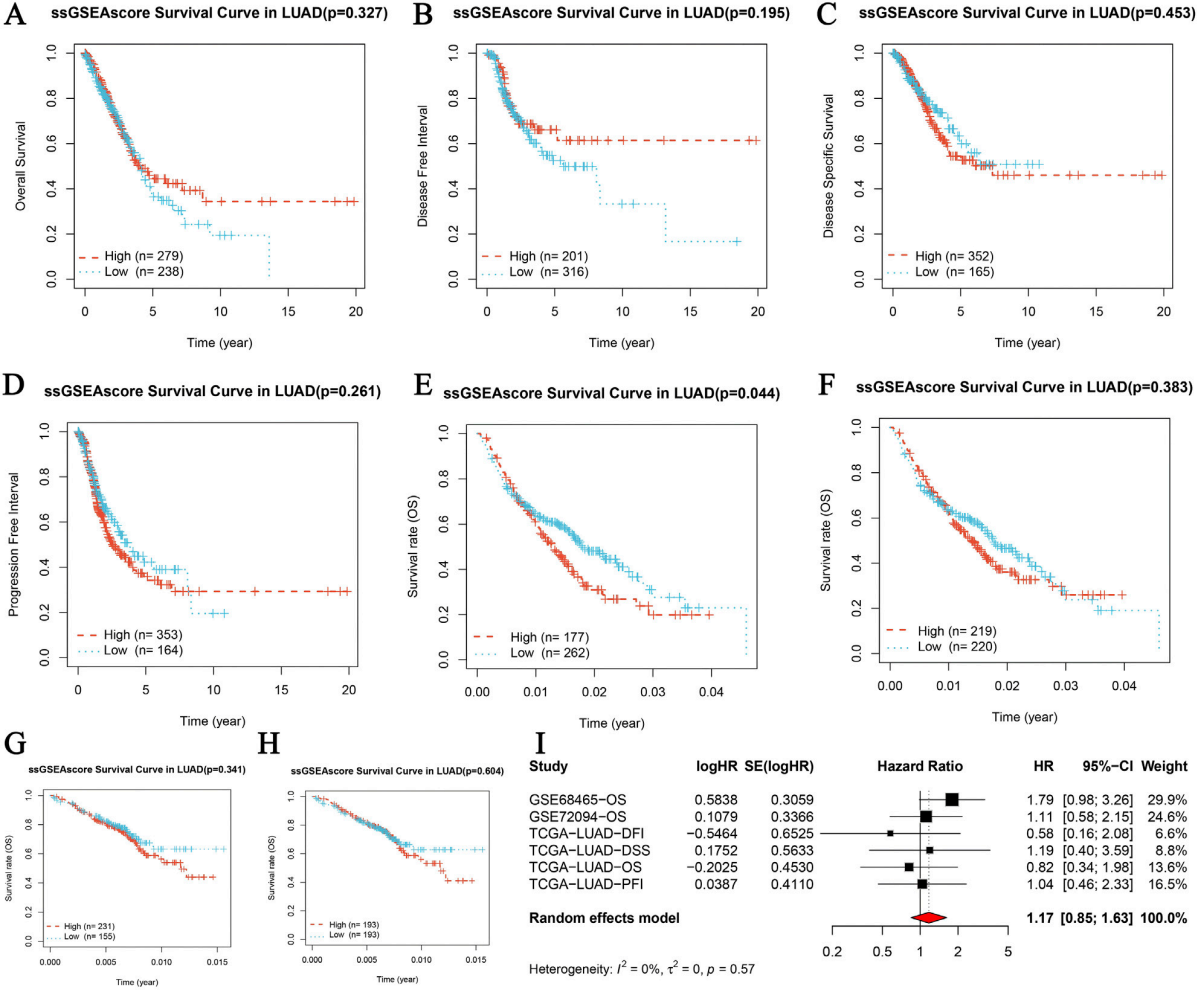
Core gene survival prognosis analysis in LUAD. **(A–D)** Kaplan-Meier survival analysis for four survival periods in LUAD, including Overall Survival (OS), Disease-Specific Survival (DSS), Progression-Free Interval (PFI), and Disease-Free Interval (DFI). Each panel represents the survival curves based on high and low expression levels of the core gene, with the number of patients indicated (high: red, low: blue). **(A)** OS survival curve (*p* = 0.327, high n = 279, low n = 314). **(B)** DSS survival curve (*p* = 0.195, high n = 201, low n = 234). **(C)** DFI survival curve (*p* = 0.453, high n = 363, low n = 378). **(D)** PFI survival curve (*p* = 0.261, high n = 353, low n = 388). **(E, F)** Overall survival curves from GSE68465 and GSE72094 datasets respectively. **(E)** GSE68465 overall survival curve (*p* = 0.044, high n = 177, low n = 222). **(F)** GSE72094 overall survival curve (*p* = 0.383, high n = 219, low n = 228). **(G–H)** Overall survival curves from GSE68465 and GSE72094 datasets, displaying Kaplan-Meier survival analysis based on high and low expression levels of the core gene. **(G)** GSE68465 overall survival curve. **(H)** GSE72094 overall survival curve. **(I)** Meta-analysis of univariate Cox survival analysis across multiple datasets, showing hazard ratios (HR) with 95% confidence intervals (CI) for each study and the combined HR using a random effects model. The plot includes studies GSE68465-OS, GSE72094-OS, TGCA-LUAD-OS, TGCA-LUAD-DSS, TGCA-LUAD-DFI, and TGCA-LUAD-PFI. The heterogeneity is indicated by I^2^ and the *p*-value.

The core gene survival prognosis analysis in LUAD was evaluated through Kaplan-Meier survival analysis and meta-analysis. Kaplan-Meier curves for Overall Survival (OS), Disease-Specific Survival (DSS), Progression-Free Interval (PFI), and Disease-Free Interval (DFI) stratified by high and low expression levels of the core gene showed no significant differences in OS (*p* = 0.327, Figure 5A), DSS (*p* = 0.195, Figure 5B), DFI (*p* = 0.453, Figure 5C), and PFI (*p* = 0.261, Figure 5D). External validation using the GSE68465 dataset revealed a significant difference in OS (*p* = 0.044, Figure 5E), while the GSE72094 dataset did not show a significant difference (*p* = 0.383, Figure 5F). A meta-analysis of univariate Cox survival analysis across multiple datasets, including GSE68465-OS, GSE72094-OS, TGCA-LUAD-OS, TGCA-LUAD-DSS, TGCA-LUAD-DFI, and TGCA-LUAD-PFI, showed a combined hazard ratio (HR) of 1.17 (95% CI: 0.85–1.48) with no significant heterogeneity (Figure 5I). These results indicate varying prognostic implications of the core gene expression in LUAD, with significant findings in some datasets and non-significant results in others, emphasizing the importance of dataset-specific factors in interpreting survival outcomes. The comprehensive analysis, including statistical evaluations and visual representations through Kaplan-Meier curves and meta-analysis plots, provides a thorough understanding of the core gene’s role in LUAD prognosis.

### Pan-cancer GSVA enrichment analysis of core genes

This study conducted a comprehensive pan-cancer GSVA enrichment analysis of core genes. It compared their expression levels across various cancer types and normal tissues using four different scoring parameters: combined z-scores, GSVA z-scores, PLAGE z-scores, and ssGSEA z-scores. Significant differences in z-scores between tumor and normal samples were observed in multiple cancer types, including GBM (Glioblastoma), THCA (Thyroid Carcinoma), PRAD (Prostate Adenocarcinoma), KIRP (Kidney Renal Papillary Cell Carcinoma), BRCA (Breast Invasive Carcinoma), KIRC (Kidney Renal Clear Cell Carcinoma), BLCA (Bladder Urothelial Carcinoma), and COAD (Colon Adenocarcinoma), indicating upregulation or downregulation of core genes in these cancers (Supplementary Figure S4). Notable differences were seen in the combined z-scores analysis, with exemplary *p*-values such as 6.1e-04 for GBM and a minute 2.6e-16 for PRAD. The GSVA z-scores depicted substantial variances in cancers like GBM and THCA, with telling *p*-values emphasizing the divergent expression of these genes. The PLAGE z-scores revealed meaningful changes in cancers like GBM and KIRC. The ssGSEA z-scores exposed consequential upregulation or downregulation of core genes in cancers such as GBM and THCA, with *p*-values showcasing statistical significance. These discoveries underscore the differing expression of core genes across diverse cancer types. They offer useful insights into their possible roles in cancer advancement and their potential usefulness as biomarkers for cancer diagnosis and treatment.

### AH-6809 exerts anti-tumor effects by modulating apoptotic and EMT pathways in LUAD

**FIGURE 6:**
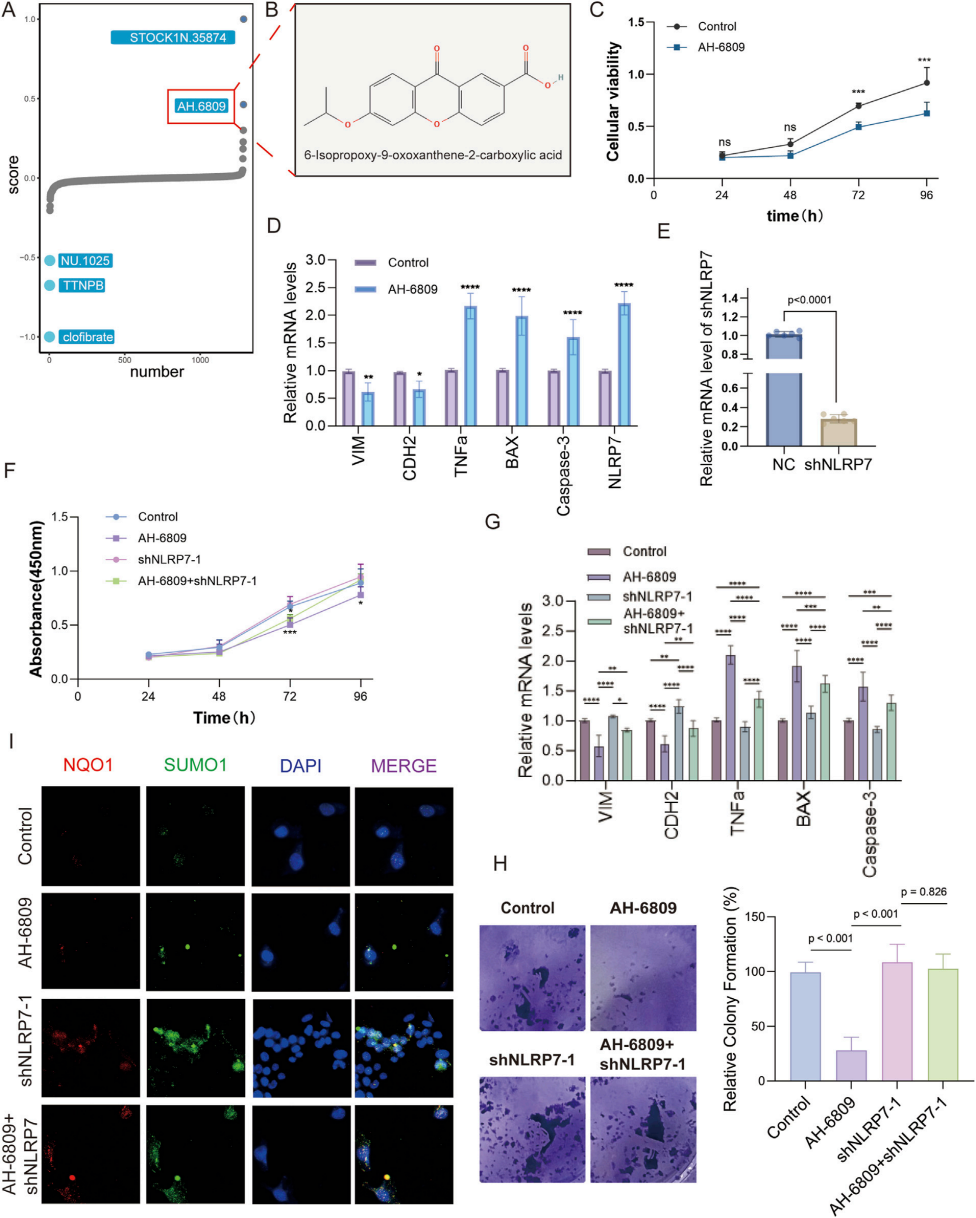
The effects of AH-6809 on cellular proliferation, apoptosis, and NLRP7 expression across various assays. **(A)** High-throughput screening identified AH-6809 as a lead compound from a drug library screen. The screening focused on compounds that influence cell proliferation, apoptosis, and inflammation, with AH-6809 selected for further study. **(B)** Chemical structure of AH-6809, identified as 6-isopropoxy-8-oxosorban-2-carboxylic acid, showcasing the molecular conformation of the compound used in the experimental assays. **(C)** Cell viability assay (CCK8) results depicting the impact of AH-6809 on cell proliferation over 96 h. Cell viability was measured at 24, 48, 72, and 96 h post-treatment. The data show a significant increase in cell viability after treatment with AH-6809, indicating a time-dependent effect. (Statistical significance: *p < 0.05, *p* < 0.01, *p < 0.001). **(D)** qRT-PCR validation of mRNA expression levels for key apoptotic and inflammatory markers (VIM, CDH2, TNFα, BAX, Caspase-3) in cells treated with AH-6809 compared to control. Treatment with AH-6809 upregulated pro-apoptotic genes and inflammatory markers significantly (*p* < 0.001), highlighting the compound’s impact on gene expression. **(E)** Confirmation of shNLRP7 knockdown efficiency. The bar graph represents a significant reduction in NLRP7 mRNA levels after transduction with shNLRP7 constructs compared to the non-targeting control (NC). The statistical significance of knockdown efficiency is shown as *p < 0.0001. **(F)** Cell proliferation assay (CCK8) assessing the phenotypic effects of shNLRP7 knockdown in the presence and absence of AH-6809. Cells with shNLRP7 knockdown showed a reduced proliferation rate, with a significant inhibitory effect observed when combined with AH-6809 treatment over 96 h (*p < 0.001). **(G)** qRT-PCR analysis showing the expression of apoptotic and EMT-related markers (VIM, CDH2, TNFα, BAX, and Caspase-3) in cells under different conditions: control, AH-6809 treated, shNLRP7 knockdown, and combined treatment of AH-6809 + shNLRP7. Combined treatment notably exacerbated the expression of apoptotic markers, while reducing EMT markers significantly (*p* < 0.01, *p < 0.001). **(H)** Colony formation assay demonstrating the effects of AH-6809 and shNLRP7 knockdown on the colony-forming ability of cells. Cells with shNLRP7 knockdown and AH-6809 treatment formed fewer colonies than controls, indicating a reduction in proliferative capacity. **(I)** Immunofluorescence analysis showing the expression of inflammatory (NQO1) and anti-apoptotic (SUMO1) markers in cells treated with AH-6809, shNLRP7, and combined treatment. The merged images show colocalization with DAPI staining, indicating significant apoptosis and inflammation in treated cells.

**FIGURE 7:**
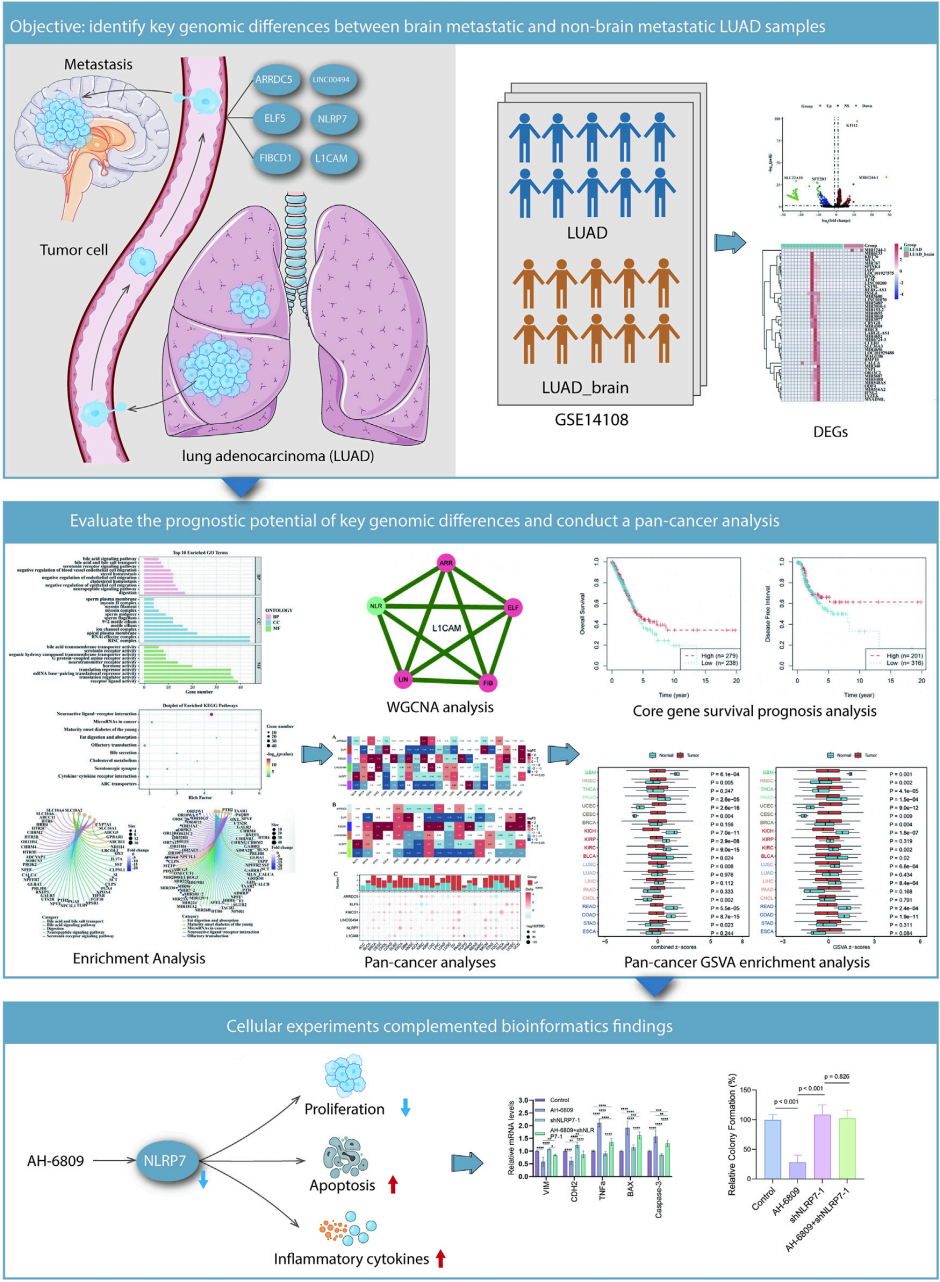
Summary of findings illustrating the interplay between genomic differences and cellular behavior in LUAD metastasis and the potential therapeutic implications of AH-6809 and NLRP7. Identification of key genomic differences between brain-metastatic LUAD and non-brain-metastatic LUAD samples. Tumor cells that metastasize to the brain are characterized by differential expression of specific genes, such as FIBCD1, ELF5, and NLRP7, identified through analysis of LUAD datasets (GSE14108). The heatmap and bar plots illustrate the significantly differentially expressed genes (DEGs) between brain metastatic and non-metastatic LUAD samples. Cellular experiments reinforce bioinformatics findings. AH-6809 treatment partially reversed the molecular changes caused by NLRP7 knockdown, suggesting that AH-6809 promotes the tumor-suppressive effects mediated by shNLRP7. The schematic illustrates the influence of AH-6809 and NLRP7 on cellular processes including proliferation, apoptosis, ROS generation, and inflammatory cytokine expression.

This investigation, concentrating on brain-metastatic LUAD and the part of NLRP7, intended to examine the molecular systems underlying the inhibitory impacts of AH-6809 on LUAD metastasis. A high-throughput screening of a chemical compound library revealed that AH-6809 significantly inhibited cancer-promoting pathways, leading to its selection for further evaluation. The initial drug screening identified AH-6809 as a compound with potential anti-cancer activity (Figures 6A,B). To assess the effects of AH-6809 on cell proliferation, a CCK8 assay was conducted. The results showed a significant reduction in cell viability following AH-6809 treatment, particularly at the 72 and 96-hour time points, indicating a time-dependent inhibition of cell proliferation (Figure 6C). To further understand the molecular effects of AH-6809, qRT-PCR was performed to measure the mRNA levels of apoptotic and epithelial-mesenchymal transition (EMT) markers in LUAD cells treated with AH-6809. The results demonstrated a significant upregulation of pro-apoptotic genes (BAX and Caspase-3) and inflammatory cytokines (TNFα), alongside a marked downregulation of EMT markers (VIM and CDH2) in AH-6809 treated cells compared to the control (*p* < 0.001) (Figure 6D). To explore the role of NLRP7 in LUAD, NLRP7 was knocked down using shRNA. Knockdown efficiency was confirmed by qRT-PCR, with a significant reduction in NLRP7 mRNA levels in shNLRP7-treated cells compared to the non-targeting control (*p* < 0.001) (Figure 6E). However, when combined with AH-6809 treatment, the inhibitory effects on cell proliferation were partially reversed, suggesting a complex interaction between AH-6809 and NLRP7-mediated pathways (Figure 6F). The colony formation assay revealed that cells with NLRP7 knockdown exhibited enhanced colony-forming ability compared to control cells, indicating that NLRP7 may promote tumorigenicity. However, AH-6809 treatment significantly reduced colony formation, and this effect was partially mitigated in the presence of shNLRP7 (Figure 6H). Immunofluorescence analysis showed altered expression of inflammatory marker NQO1 and anti-apoptotic marker SUMO1 following treatment with AH-6809, shNLRP7, and their combination. Colocalization of these markers with DAPI revealed significant increases in apoptosis and inflammation in cells treated with AH-6809, particularly in combination with shNLRP7, indicating that these proteins may be involved in the molecular mechanisms of AH-6809’s anti-cancer activity (Figure 6I). In conclusion, this study demonstrates that AH-6809 exerts potent anti-tumor effects in LUAD by modulating key apoptotic and EMT-related pathways. AH-6809 likely reverses the molecular characteristics induced by NLRP7 dysregulation, promoting tumor-suppressive effects (Figure 7). These findings suggest that targeting NLRP7 with AH-6809 could be a promising therapeutic approach for treating brain-metastatic LUAD.

## Discussion

We sought to study the rich gene network in LUAD metastasis and discovered six significant players: ARRDC5, ELF5, FIBCD1, LINC00494, NLRP7 and L1CAM, all of which are ribonucleic acids that direct migration as well (Yao et al., 2020; Zhang et al., 2020). We took an additional step and carried it out in a pan-cancer analysis view. With this approach, the expression dynamics of each of these genes across multiple cancer types is given and examined in depth (Mishra et al., 2016; Anaya et al., 2016). Their potential as prognostic markers was also described.

The expression levels of these genes were significantly associated with the metastatic potential in LUAD according to our study. In particular, increased expression of ARRDC5 and FIBCD1 or ELF5 provided predictive value for higher risk to develop metastasis both in non-IMIs AND IMIs studies which were consistent with what we had got from the final prognostic model. Our pan-cancer analysis further substantiated these findings, demonstrating that the downstream effects of CCDC6 and PTK2 on cell function are likely pervasive in cancer beyond just HL, a finding consistent with prior evidence showing their broader roles in cancer progression. ARRDC5 and ELF5 were ubiquitously related to bad prognosis in various cancer types such as represented by the example of ARRDC5 (43,44), but also grouped together, indicative that they may be novel universal prognostic markers. These findings are consistent with previous work suggesting that ARRDC5 and ELF5 contribute to cancer promotion. Our study, however takes this knowledge further up by showing the pan-cancer relevance of these genes and possible importance as prognostic markers (Anaya et al., 2016; Nagy et al., 2021). Importantly, the opposite correlation found with L1CAM in metastatic LUAD as it was previously thought of to facilitate migration and invasion by cancer cells in other cancers indicates that its role can be quite complex on a case-by-case basis within our current understanding (Hai et al., 2012; Tischler et al., 2011).

Several genes, including ARRDC5, ELF5, FIBCD1, LINC00494, NLRP7, and L1CAM, have been implicated in various aspects of cancer progression and metastasis (Altevogt et al., 2020; Graca et al., 2022; Luk et al., 2018). However, their specific roles in LUAD, particularly in brain metastasis, have not been fully elucidated. On one hand, the pivotal role of NLRP7 in inflammation and cancer immunity, as well as association with tumor metastasis is revealed (Feng et al., 2022); on the other, L1CAM was reported to get involved in tumor invasiveness across a number of malignancies except for LUAD metastases which remains controversial (Wang et al., 2022).

NLRP7 plays a complex and diverse role in tumors, particularly in brain metastasis of LUAD (Lin et al., 2021; Li et al., 2021). Our experimental results demonstrate that the expression of NLRP7 is significantly lower in LUAD patients with brain metastasis compared to non-brain metastasis patients, suggesting a potential negative correlation between NLRP7 expression and the ability of lung cancer cells to penetrate the blood-brain barrier. Further gene silencing experiments revealed that treatment with AH-6809 significantly inhibited cell proliferation and induced apoptosis, an effect reversed in NLRP7 knockout cells, indicating that NLRP7 plays a crucial role in regulating tumor cell proliferation and apoptosis. Thus, NLRP7 may inhibit brain metastasis by regulating cell cycle and apoptosis pathways (Reynaud et al., 2023). Moreover, AH-6809, as a potential activator of NLRP7, could inhibit LUAD brain metastasis by enhancing NLRP7’s function. Collectively, NLRP7 is a key regulator of tumor cell survival and migration, and targeting its expression or activity could represent a novel therapeutic strategy against LUAD brain metastasis.

As a member of the NOD-like receptor family, NLRP7 exhibits complex functions in various tumors (Li et al., 2021). It regulates inflammation, tumor cell proliferation, apoptosis, and the immune microenvironment. In ovarian cancer, NLRP7 influences cancer cell survival by regulating apoptosis and autophagy, with low expression linked to tumor progression and invasiveness (Mamoor, 2020). NLRP7 also appears to regulate the stress response and drug resistance in ovarian cancer cells (Mamoor, 2020). In gastric cancer, NLRP7 expression correlates closely with tumor grading and prognosis, with low levels indicating increased invasiveness and poor outcomes, suggesting its potential as a prognostic marker (Jiang et al., 2017). In breast cancer, NLRP7 affects tumor progression and metastasis by regulating immune cell infiltration and inflammation (Ershaid et al., 2019). Activating NLRP7 may restore immune balance in the tumor microenvironment, thereby inhibiting metastasis (Lee et al., 2020). Low expression of NLRP7 has been linked to enhanced anti-apoptotic abilities in pancreatic cancer, making it a potential therapeutic target.

Meanwhile, SUMO1 is a key player in post-translational protein modification via SUMOylation, regulating protein function, localization, and stability (Barry and Lock, 2011). In tumors, SUMOylation critically regulates cell proliferation and apoptosis signaling (Gong et al., 2017). Our findings show that SUMO1 expression is significantly upregulated in AH-6809-treated LUAD cells, suggesting that AH-6809 may regulate apoptosis via SUMOylation, thereby inhibiting tumor growth (Ke et al., 2019). This may be connected to sustained activation of nuclear signaling pathways like NF-κB and STAT3, which govern cell survival and apoptosis (Fan et al., 2013). Thus, SUMO1-mediated post-translational modification is crucial in regulating tumor cell fate. Additionally, AH-6809 was shown to enhance antioxidant defenses by upregulating NQO1, maintaining cellular homeostasis and resisting stress responses. Overall, by regulating SUMOylation and antioxidant defense mechanisms, AH-6809 presents a potential therapeutic strategy for LUAD, warranting further clinical investigation.

Over the past few years, as novel targeted therapeutic strategies have emerged, it has become theoretically possible to improve treatment efficacy and reduce toxicity, hence revolutionize precision medicine (Vargas-Sierra et al., 2024). Systematic reviews and meta-analyses have become more popular since their first applications in biomedical research, encompassing different methodologies not only within drug development but also including studies carried out using bioinformatical approaches (Wu et al., 2024). They have been widely recognized, not only for *in vivo* and clinical applications but also those of basic research and translational medicine. The development course of computer-aided drug design has also attracted our attention to this discipline, which created some new research hotspots and provided more chances for novel drugs exploring (Wu et al., 2024). In contrast, the role of cell death and metabolic regulation in facilitating disease progression has garnering increased prominence over the same period (Lin et al., 2023), providing novel drugable targets. Much of the increased efficacy and specificity of treatments, for example, by targeting specific proteins or genetic pathways as outlined in this report (Hong et al., 2024). For example, kiwi root extract exhibits gastric cancer inhibitory effect by suppressing Wnt/β-catenin pathway (Chu et al., 2023). The combination of modern technology with traditional Chinese medicine also offers new perspectives and potential for drug development (Wang et al., 2023). Significant advancements in materials science have enabled the application of various novel composite materials in biomedical and engineering fields (Wu et al., 2024). Additionally, applying photothermal therapy and nanoparticle-based drugs derived from natural substances in regulating the microenvironment and alleviating inflammation has shown promising future directions for treatment (Shi et al., 2023; Yang et al., 2023). The development of “off-the-shelf” gene therapy nanoparticles, particularly in orthopedics and soft tissue repair, has opened up new clinical applications in regenerative medicine (O’Shea et al., 2024). Studies have shown that by improving drug delivery systems and utilizing nanotechnology, drug targeting and therapeutic efficacy have been significantly enhanced (Romanovska et al., 2024). These achievements not only provide new insights and approaches within their respective fields but also demonstrate the immense potential of interdisciplinary collaboration in disease diagnosis and treatment, highlighting the importance of integrated data analysis and multidimensional evaluation in modern medicine (Oinaka et al., 2024; Latini et al., 2024). Furthermore, social support has a significantly positive impact on the mental health of cancer patients, a finding that has been well-documented, particularly in studies conducted in China (Zhu, 2024; Paillard-Brunet and Couillet, 2024).

Identifying genes that play crucial roles in the development of LUAD metastasis and have predictive power across multiple cancers will help enable personalized medicine (Li et al., 2018; Liu et al., 2023). Molecular typing for metastasis and prognostication of patients, based on gene expression profiling, is crucial in guiding personalized therapeutic strategies for individuals (Bustin and Dorudi, 2004; Van’T Veer et al., 2002). Our findings also provide new insights into how these genes impact cancer progression, focusing in particular on how they control the clinical environment of the tumour and human immune responses. They give a new perspective for future therapeutic research (Yang et al., 2023; Andrews et al., 2018). Seeing as our research was based on a retrospective study and its data of shares notched a relatively limited sample size, there may be potential bias in what we have found (Lin, 2018; Zhou, 2014). In addition, our use of publicly available databases may restrict the detail of our findings even though it yields valuable data from something broader in scope (Altamimi et al., 2024; Phillippi et al., 2017). Potential sources of bias exist, such as differences in data collection methods, variations in patient populations, and possible lack of control over data quality. This is why further experimental studies will be needed in order to determine exactly the functional roles these genes are playing in LUAD metastasis (Wang et al., 2020; Cheng et al., 2021). These studies will be larger and more diversified cohorts than ours were, confirming our findings at the same time opening up clinical applications for these genes as biomarkers and drug targets (Minn et al., 2005; Yao et al., 2020). Therefore, future research can adopt studying different patient populations, exploring additional datasets from other sources, and applying the same analytical methods to different cohorts to evaluate the consistency of our findings in different environments. In addition, in-depth *in vitro* experiments and additional *in vivo* animal experiments are the focus of the next research step.

Without a doubt, our investigation sheds light on the intricate gene interactions involved in LUAD metastasis and broadens this inquiry to a pan-cancer examination. Through comprehensive genomic, epigenomic, and transcriptomic dissections, we pinpointed ARRDC5, ELF5, FIBCD1, LINC00494, NLRP7, and L1CAM as possible prognostic indicators across multiple cancer types (Zhao et al., 2015; Zhu et al., 2017). This discovery significantly advances our comprehension of cancer progression (Paltridge et al., 2013; Nicolini, 2023). Despite the restrictions of our study, it establishes a solid foundation for future research and has the potential to advise clinical practice, in the end adding to improved patient care and results (Jeffs et al., 2018; Ferrill et al., 2021). Supplementary studies are warranted to substantiate these conclusions and scrutinize their therapeutic potential, paving the way for more effective and individualized cancer treatment strategies (Ledermann et al., 2015; Kamel and Al-Amodi, 2017).

## Conclusion

In this study, NLRP7, ELF5, FIBCD1, ARRDC5, LINC00494, and L1CAM were identified as key prognostic markers for LUAD metastasis, particularly brain metastasis. Among these, NLRP7 was highlighted as a critical regulator of metastatic progression. The therapeutic potential of AH-6809 was demonstrated through its ability to inhibit LUAD cell proliferation, induce apoptosis, and modulate key molecular pathways such as SUMO1-mediated post-translational modifications and NQO1 expression. These findings suggest that targeting NLRP7 and related pathways could offer new strategies for preventing LUAD metastasis. Further experimental validation and clinical studies are needed to confirm these results and explore their therapeutic applications.

## Data Availability

The original contributions presented in the study are included in the article/Supplementary Material, further inquiries can be directed to the corresponding author.
