# Supplementary material for: AH-6809 mediated regulation of lung adenocarcinoma metastasis through NLRP7 and prognostic analysis of key metastasis-related genes
Source: Front Pharmacol. 2024 Dec 4;15:1486265. doi: 10.3389/fphar.2024.1486265 (PMC11652142; doi:10.3389/fphar.2024.1486265)
Supplement: Supplementary file 1 [file DataSheet1.docx]

**Supplementary Figures**


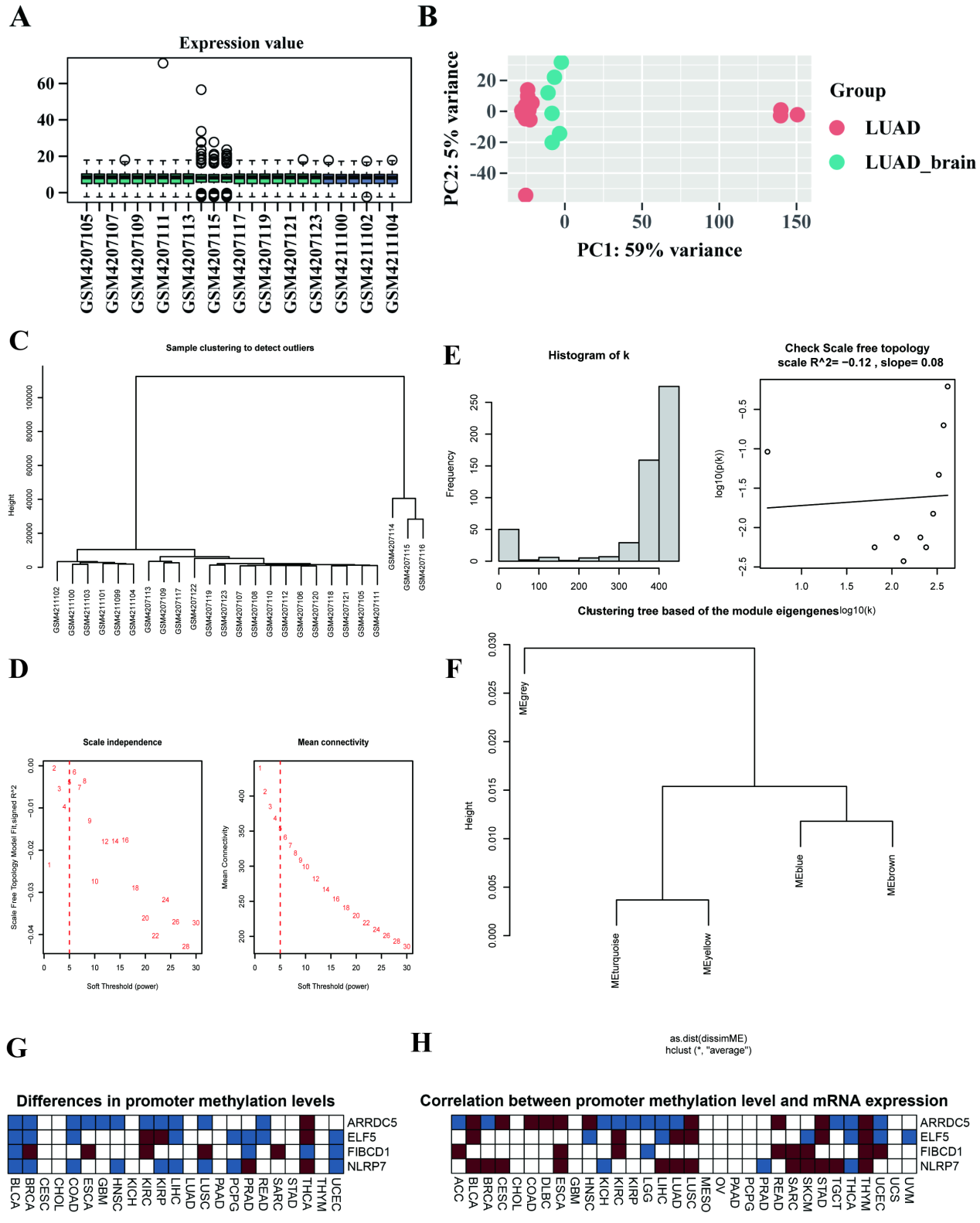


**Supplementary Figure 1. Analysis of gene expression, methylation, and clustering in LUAD and brain metastasis samples.**

(A) Normalized box plot of expression values for LUAD primary tumors and LUAD brain metastasis samples. Expression data from GSE141685 were utilized, comparing primary LUAD tumors to brain metastasis samples. The plot shows distribution of gene expression levels across different samples, indicating variations between primary and metastatic tissues.

(B) Principal Component Analysis (PCA) of the gene expression data for LUAD primary tumors and brain metastasis samples.

(C) Hierarchical clustering dendrogram based on gene expression profiles of LUAD and brain metastasis samples. The dendrogram is used to identify outliers in the dataset and visualize sample clustering based on expression similarities. Outlier samples are excluded from downstream analysis to ensure data quality.

(D) Scale independence and mean connectivity plots, showing the degree of scale-free topology and network connectivity for the gene co-expression network analysis. These metrics confirm the network’s adherence to scale-free properties, crucial for understanding the biological relevance of the gene interactions.

(E) Histogram of node connectivity (k) within the co-expression network. This plot displays the distribution of connectivity values across the network nodes, with highly connected nodes (hubs) playing key roles in the network’s structure.

(F) Dendrogram of hierarchical clustering based on module eigengenes. The clustering tree groups genes into modules based on their co-expression profiles, revealing modules of highly correlated genes. This clustering provides insights into the underlying structure of the gene co-expression network in LUAD and brain metastasis samples.

(G-H) Heatmap showing differences in promoter methylation levels for core genes (AARDOCS, ELF5, FIBCD1, NLRP7) across LUAD and brain metastasis samples. Each cell represents methylation status (blue for lower methylation, red for higher methylation), revealing significant variations in methylation across different samples.

**
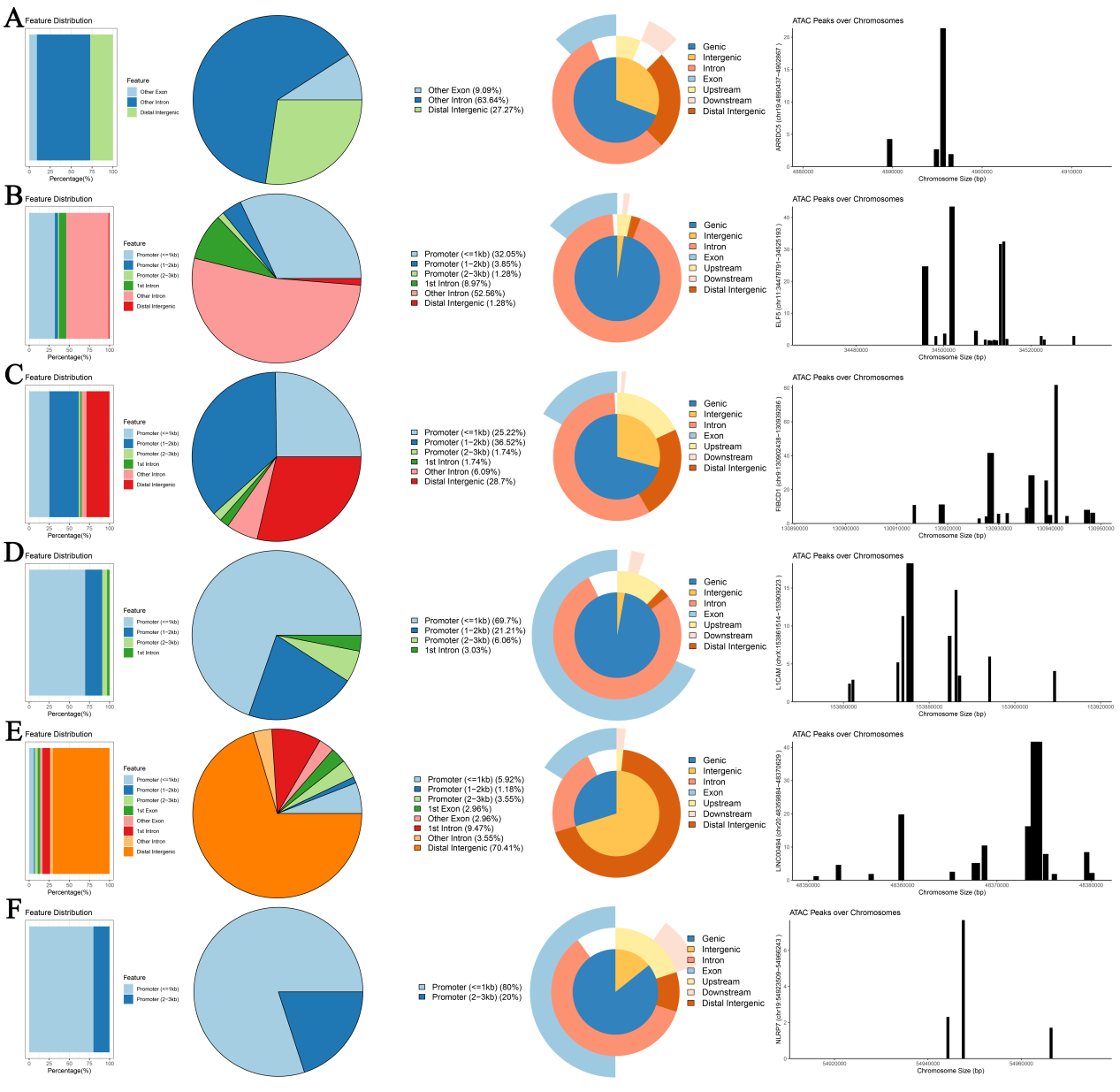
**

**Supplementary Figure 2. Core gene promoter methylation analysis.**

(A) ARRDC5 Promoter Methylation Analysis: The bar chart represents the average distribution of methylation levels across the ARRDC5 promoter region, indicating the percentage of promoter hypermethylation and hypomethylation. The circos plot depicts methylation distribution between CpG islands, gene body, promoter regions and distal intergenic regions from birth to old age in human brain. Naturally, the methylation peaks and their values are shown along the ARRDC5 promoter (the right-most plot).

(B) ELF5 Promoter Methylation Analysis: What the bar chart indicates is the methylation levels in the ELF5 promoter region. The pie chart breaks down the samples according to their methylation point. The circos plot shows the distribution of methylation along the genome and the plot on the right represents peaks of methylation levels across ELF5 promoter.

(C) FIBCD1 Promoter Methylation Analysis: The bar chart describes the extent of methylation in the FIBCD1 promoter. The pie chart displays how the samples are distributed according to their methylation status. The circos plot displays the genomic distribution of methylation, while the rightmost plot focuses on both where and what concentration FIBCD1 is being methylated.

(D) L1CAM Promoter Methylation Analysis: As you can see from the bar chart, it reflects methylation degrees of the L1CAM promoter district. Meanwhile, pie diagrams are used to depict frequencies for different mining categories (see Figure below). Contraction Of Cancer Volume In All Directions Under High-Resolution Circos Technology That ring chart displays genomic characteristics along with the methylation biased, longest although not least, within this plot we can see large number diagrams to locate small peaks which are methylation sites in L1CAM Promote.

(E) The bar chart indicates the methylation levels of the LINC00494 promoter. Presented as a pie chart, the plot shows clearly what percentage of tested samples had methylation--as they say "positive" or "negative" may not interest you but it can later go off in Venn diagram and help further refine your search for new sets of genes that are closely related to each other different aspects complete picture can achieve more comprehensive results quite rapidly. In the circos plot, various methylations are shown at genomic level. The rightmost plot illustrates page contains a number of methylation sites, which are represented by circles and peaks on the LINC00494 promoter in relation with intensity.

(F) NLRP7 Promoter Methylation Analysis: The bar chart represents methylation levels in the NLRP7 promoter. The pie chart shows sample distribution by methylation status. The circos plot details the genomic distribution of methylation, and the rightmost plot presents the concentration and distribution of methylation peaks in the NLRP7 promoter.


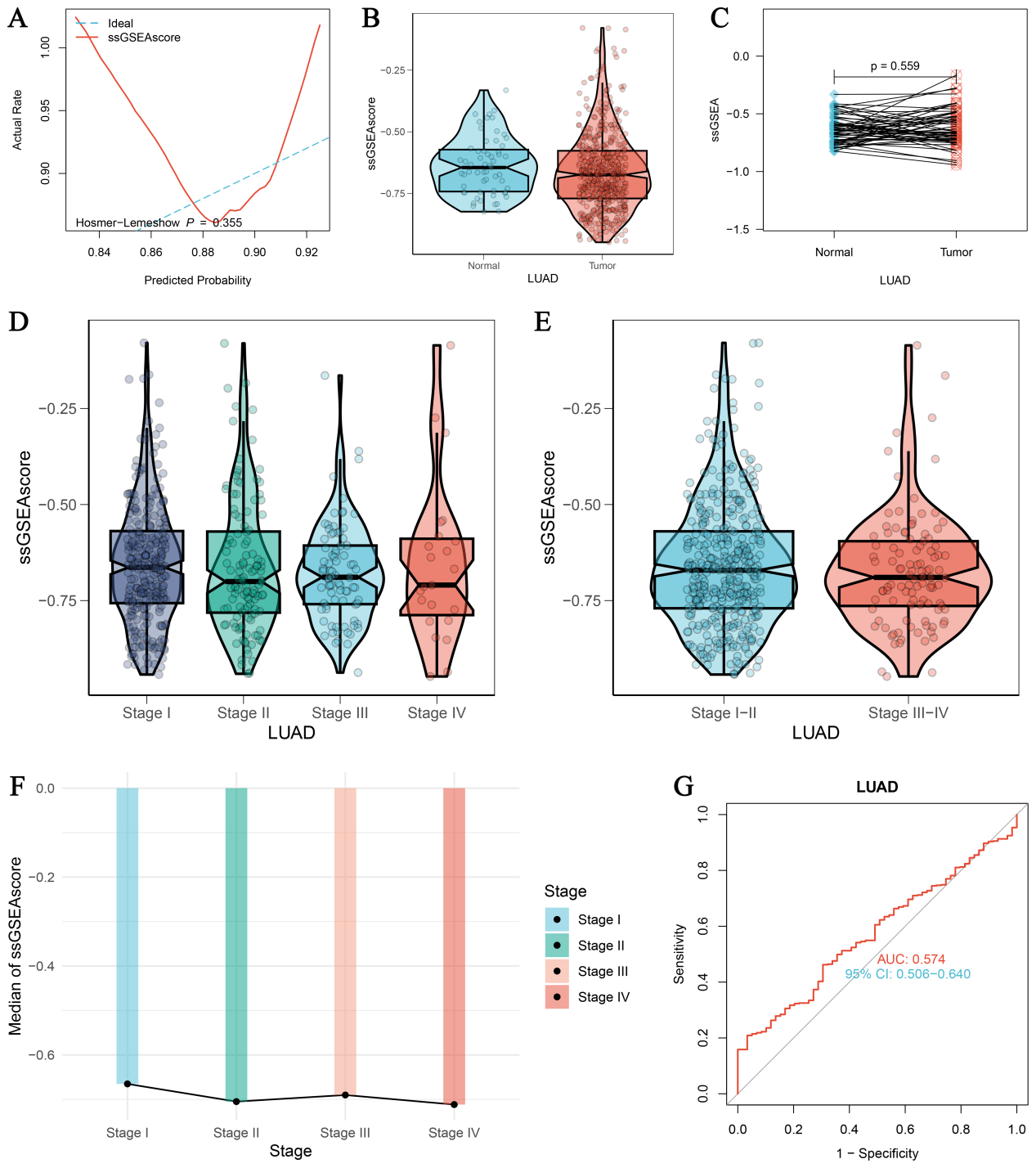


**Supplementary Figure 3. Correlation of core gene expression with LUAD prognosis.**

(A) Calibration curve and goodness-of-fit test for ssGSEAscore expression in predicting tumor versus normal group. The plot compares the predicted probability of the ssGSEAscore with the actual rate, with the Hosmer-Lemeshow test result (P = 0.555) indicating the model fit.

(B) Violin plot showing the differential expression of ssGSEAscore between normal and LUAD tumor groups, illustrating higher scores in the tumor group.

(C) Paired difference analysis of ssGSEAscore expression between normal and tumor tissues. Each line represents paired samples, with no significant difference observed (p = 0.559).

(D) Violin plot depicting ssGSEAscore expression across the four clinical stages of LUAD, from Stage I to Stage IV. The data indicate variability in ssGSEAscore with disease progression.

(E) Violin plot comparing ssGSEAscore expression between early-stage (Stage I-II) and late-stage (Stage III-IV) LUAD, showing significant differences.

(F) Median ssGSEAscore values across the four stages of LUAD, with a line plot overlay indicating the trend expression with disease progression.

(G) Receiver operating characteristic (ROC) curve evaluating the diagnostic efficacy of ssGSEAscore in distinguishing tumors from normal tissues in LUAD patients. The area under the curve (AUC) is 0.574 with a 95% confidence interval of 0.506-0.640.


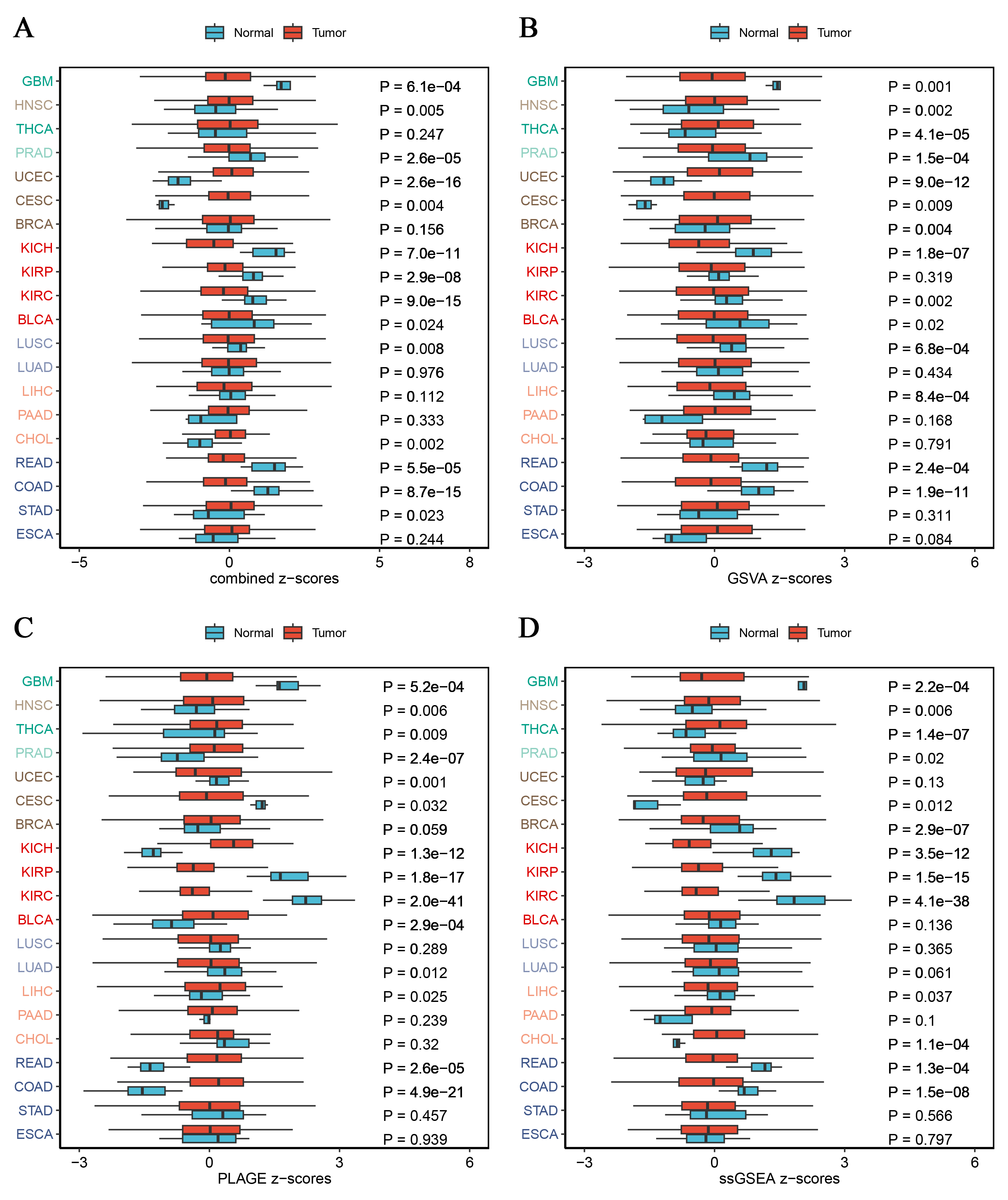


**Supplementary Figure 4. Pan-cancer GSVA enrichment analysis of core genes.**

1. D) Box plots illustrating the comparison of GSVA scores for core genes across various cancer types and normal tissues using four different scoring parameters. (A) Combined z-scores for the core genes in different cancer types compared to normal tissues. Significant differences in z-scores between tumor and normal samples are observed in multiple cancer types, including GBM, THCA, PRAD, KIRP, BRCA, KIRC, BLCA, and COAD, indicating upregulation or downregulation of core genes in these cancers. Statistical significance is indicated by p-values next to each cancer type. (B) GSVA z-scores showcasing the enrichment of core genes in various cancer types versus normal tissues. Notable differences are seen in cancers such as GBM, THCA, PRAD, KIRP, KIRC, and BLCA, with significant p-values highlighting the differential expression of these genes. (C) PLAGE z-scores representing the differential expression of core genes in tumor versus normal tissues. Significant changes are observed in cancers including GBM, THCA, PRAD, KIRP, KIRC, and BLCA. P-values indicate the level of statistical significance for each comparison. (D) ssGSEA z-scores comparing core gene expression between different cancers and normal tissues. Significant upregulation or downregulation of core genes is detected in cancers such as GBM, THCA, PRAD, KIRP, KIRC, and BLCA, with p-values demonstrating the statistical significance of these differences.
